# B Cell Activation by Outer Membrane Vesicles—A Novel Virulence Mechanism

**DOI:** 10.1371/journal.ppat.1000724

**Published:** 2010-01-15

**Authors:** Maria Laura A. Perez Vidakovics, Johan Jendholm, Matthias Mörgelin, Anne Månsson, Christer Larsson, Lars-Olaf Cardell, Kristian Riesbeck

**Affiliations:** 1 Medical Microbiology, Department of Laboratory Medicine, University Hospital Malmö, Lund University, Malmö, Sweden; 2 Section of Clinical and Experimental Infectious Medicine, Department of Clinical Sciences, Lund University, Lund, Sweden; 3 Division of ENT Diseases, Department of Clinical Sciences, Intervention and Technology, Karolinska Institute, Huddinge, Sweden; 4 Center for Molecular Pathology, Department of Laboratory Medicine, University Hospital Malmö, Lund University, Malmö, Sweden; University of Southampton, United Kingdom

## Abstract

Secretion of outer membrane vesicles (OMV) is an intriguing phenomenon of Gram-negative bacteria and has been suggested to play a role as virulence factors. The respiratory pathogens *Moraxella catarrhalis* reside in tonsils adjacent to B cells, and we have previously shown that *M*. *catarrhalis* induce a T cell independent B cell response by the immunoglobulin (Ig) D-binding superantigen MID. Here we demonstrate that *Moraxella* are endocytosed and killed by human tonsillar B cells, whereas OMV have the potential to interact and activate B cells leading to bacterial rescue. The B cell response induced by OMV begins with IgD B cell receptor (BCR) clustering and Ca^2+^ mobilization followed by BCR internalization. In addition to IgD BCR, TLR9 and TLR2 were found to colocalize in lipid raft motifs after exposure to OMV. Two components of the OMV, i.e., MID and unmethylated CpG-DNA motifs, were found to be critical for B cell activation. OMV containing MID bound to and activated tonsillar CD19^+^ IgD^+^ lymphocytes resulting in IL-6 and IgM production in addition to increased surface marker density (HLA-DR, CD45, CD64, and CD86), whereas MID-deficient OMV failed to induce B cell activation. DNA associated with OMV induced full B cell activation by signaling through TLR9. Importantly, this concept was verified *in vivo*, as OMV equipped with MID and DNA were found in a 9-year old patient suffering from *Moraxella* sinusitis. In conclusion, *Moraxella* avoid direct interaction with host B cells by redirecting the adaptive humoral immune response using its superantigen-bearing OMV as decoys.

## Author Summary

Outer membrane vesicles secreted by pathogenic bacteria are recognized as a long-distance delivery system which transports diverse virulence factors, and allows pathogens to interact with the host, and hence the possibility to modify the immune response without close contact. Our study shows that *Moraxella catarrhalis* outer membrane vesicles that also exist in patients can activate human B cells isolated from pharyngeal lymphoid tissue. These findings have significant implications for understanding *Moraxella* pathogenesis since palatine tonsils are a potential tissue reservoir. Vesicles secreted by *Moraxella* bind to tonsillar B cells through the superantigen *Moraxella* IgD-binding protein designated MID. The interaction between MID and the B cell receptor induces Ca^2+^ mobilization and receptor clustering in lipid raft motifs followed by internalization of vesicles. Mainly Toll-like receptor 9, a pathogen recognition receptor of the innate immune system, participates in the signaling induced by vesicles through sensing of DNA associated with vesicles. The vesicle-dependent B cell activation induces up-regulation of surface activation markers in addition to IL-6 and IgM secretion. Vesicle secretion provides *Moraxella* with a sophisticated mechanism to modify the host immune response, avoiding contact between bacteria and the host.

## Introduction

*Moraxella catarrhalis* is one of the major respiratory pathogens in humans causing acute otitis media in children, sinusitis and laryngitis in adults as well as exacerbations in patients diagnosed with chronic obstructive pulmonary disease (COPD) [1],[2]. The *M*. *catarrhalis* carriage varies during life from very high in young children to low in healthy adults. Recent findings that *M*. *catarrhalis* could hide intracellularly and the fact that biofilm forming bacteria like *Moraxella* are easily overlooked in swab samples suggest that the overall colonization of *M*. *catarrhalis* could be underestimated [3]–[5]. A study of pharyngeal lymphoid tissue using fluorescent *in situ* hybridization (FISH) has shown that 91% of the adenoids and 85% of the palatine tonsils harbour *M*. *catarrhalis* [6]. It was also demonstrated that *M*. *catarrhalis* colocalizes with B cells in the outer mantel zone of the lymphoid follicles. Thus, these observations in human tonsils explain where the non-invasive pathogen *M*. *catarrhalis* may interact with B cells.

*M*. *catarrhalis* interferes with the immune system in several ways [7]. One of its most intriguing interactions is the IgD-binding capacity (for a review see [8]). The outer membrane protein and superantigen *Moraxella* immunoglobulin (Ig) D binding protein (MID) is a trimeric autotransporter [9] and the IgD-binding domain is located within amino acids 962-1200 (MID962-1200) [10]. MID binds to amino acids 198–224 in the C_H_1 region on human IgD [11] and this non-immune cross-linking explains the mitogenic effect of *M*. *catarrhalis* on IgD^+^ human B cells [12]. Cross-linking of the BCR leads to receptor-mediated endocytosis of whole bacteria and a lower threshold for pathogen recognition receptor (PRR) signalling in the B cell [13]. Toll-like receptor (TLR) 9 is the most important PRR in *M*. *catarrhalis*-induced B cell activation, but TLR1, TLR2 and TLR6 also contribute to the activation. B cell activation by *M*. *catarrhalis* leads to a polyclonal IgM production, suggesting a delayed production of protective antibodies [12],[14].

All Gram-negative bacteria naturally release outer membrane vesicles (OMV) during both planktonic growth and in surface-attached biofilm communities [15]. These spherical bilayered OMV are liberated from the outer membrane and range in size from 50–250 nm in diameter. OMV produced by pathogenic bacteria contain adhesins, invasins and immunomodulatory compounds such as lipopolysaccharide (LPS) (for a review see [16],[17]). Production of OMV represents a distinct secretion mechanism that allows bacteria to release and disseminate a large, complex group of proteins and lipids into the extracellular milieu. Several studies have demonstrated that OMV play a role as protective transport vesicles, delivering toxins, enzymes and DNA to eukaryotic cells as well as being key factors in natural competence [18]–[22]. OMV can also improve bacterial survival in the host by directly mediate bacterial binding and invasion, causing cytotoxicity, and modulating the host immune response [23],[24]. By acting as decoys to the immune system, OMV may enable bacteria to evade immune detection during colonization, binding and removing cell-targeted bactericidal factors. We have previously shown that OMV from *M*. *catarrhalis* contribute to an increased survival of *Haemophilus influenzae* in human serum by binding and neutralizing C3 *in vitro* [25]. Thus, based upon several lines of evidence it is clear that OMV produced by colonizing pathogens have a complex and as yet unexplored impact on the immune response. In this study, we examined the capacity of OMV to interact with human B cells isolated from pharyngeal lymphoid tissues, where *M*. *catarrhalis* can reside [6], and in detail studied the virulence factors and mechanisms involved in this process.

## Results

### *M*. *catarrhalis* that are endocytosed by human tonsillar B lymphocytes do not survive intracellularly

**Figure 1:**
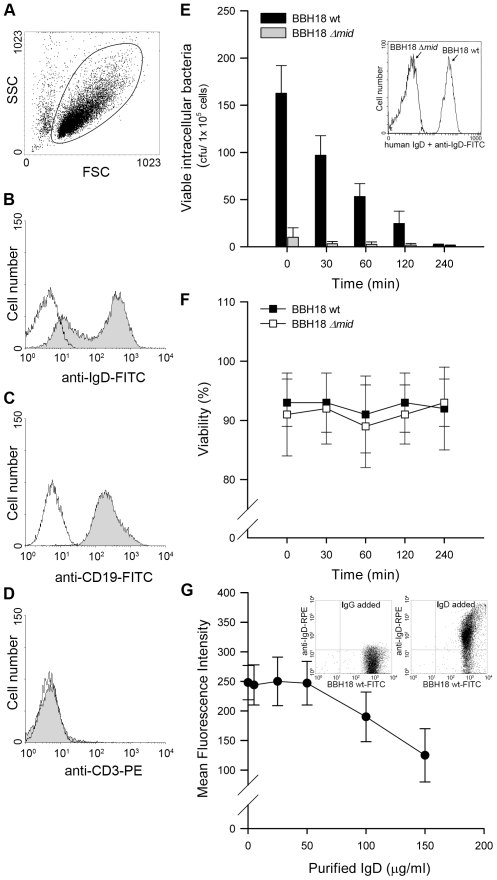
Internalized MID-expressing *M*. *catarrhalis* do not survive in tonsillar B cells. (A) A representative dot plot displaying forward and side scatters of the purified tonsillar B cells used for live-cell gating. Each preparation of purified tonsillar B lymphocytes were analysed as routine control by flow cytometry for IgD (B), CD19 (C), and CD3 (D) expression. White profiles correspond to the isotype control Abs used as negative controls. Representative flow cytometry plots are shown. (E) *M*. *catarrhalis* wild type (BBH18 wt) or an isogenic MID-deficient mutant (BBH18 Δ*mid*) were incubated with purified tonsillar B lymphocytes for 1 h. Thereafter, B cells were washed and treated with gentamicin to kill extracellular bacteria followed by thorough washes. At the indicated times, infected cells were lysed mechanically and plated on agar plates. Colony forming units (cfu) were counted after incubation for 24 h at 37°C. The mean values of three separate experiments with different donors are demonstrated. Error bars indicate SEM. Insert shows IgD binding to *M*. *catarrhalis* BBH18 wt and the MID-deficient mutant BBH18 Δ*mid*. Bacteria were grown on solid medium overnight. After incubation with a human IgD standard serum followed by a FITC-conjugated anti-human IgD pAb and several washings, bacteria were analyzed by flow cytometry. (F) The viability of B cells infected with *M*. *catarrhalis* BBH18 wt and BBH18 Δ*mid* was assessed at the indicated times by trypan blue exclusion staining. The mean values of three independent experiments are demonstrated. Error bars indicate SEM. (G) The capacity of purified human IgD to block *M*. *catarrhalis* binding to B cells was analyzed by flow cytometry. FITC-labelled *M*. *catarrhalis* wild type were treated with increased concentrations of purified human IgD, washed and incubated with human B cells for 30 min at 37°C. After several washes, bacterial binding to B cells were analyzed by flow cytometry. The mean values of three independent experiments are demonstrated. Error bars indicate SEM. Insert shows IgD-binding to FITC-labelled *M*. *catarrhalis*. Bacteria were incubated with a human IgG standard (50 µg/ml) (left panel) or purified IgD (50 µg/ml) (right panel). After several washes, *Moraxella* were incubated with an RPE-conjugated anti-human IgD mAb and analyzed by flow cytometry.

*M*. *catarrhalis* can be found adjacent to B cells in tonsils [6] and we have recently shown that this pathogen is internalized by tonsillar B cells [13]. Intracellular survival of bacteria in B cells would thus be a potential escape mechanism in the host. To test this hypothesis, we isolated B cells from human tonsils by negative selection. To ensure the purity of the B cell preparation, all isolated lymphocytes were screened for CD3, CD19, and IgD expression by flow cytometry (Fig. 1A–D). Tonsillar B cells were incubated with *M*. *catarrhalis* BBH18 (Table 1) or an isogenic mutant deficient in the IgD-binding protein MID (BBH18 Δ*mid*) for 1 h. Extracellular bacteria were killed by gentamicin in a conventional survival assay and the intracellular fate was assessed at different time points. Interestingly, intracellularly residing bacteria were rapidly killed by B lymphocytes suggesting that this cell type would not be a reservoir for *Moraxella* (Fig. 1E). In contrast, non-IgD binding bacteria as demonstrated with the mutant *M*. *catarrhalis* BBH18 Δ*mid* were not taken up by B cells as compared to the MID-expressing wild type. The MID-expressing wild type *Moraxella* bound soluble IgD, whereas the MID-deficient mutant did not as revealed by flow cytometry analysis (Fig. 1E; insert). Finally, B cell viability was not affected by the presence of *M*. *catarrhalis* at the different time points evaluated (Fig. 1F).

**Table 1:** *Moraxella catarrhalis* strains used in the present study.

Strain	Phenotype	Reference
**BBH18 wt**	Clinical isolate, wild type	[42]
**BBH18 Δ** ***mid***	MID-deficient	[67],[68]
**BBH18 Δ** ***uspA1***	UspA1-deficient	[60]
**BBH18 Δ** ***uspA2***	UspA2-deficient	[60]
**BBH18 Δ** ***uspA1/A2***	UspA1/A2-deficient	[60]
**BBH18 Δ** ***mid/uspA1/2***	MID and UspA1/A2-deficient	[60],[67]
**KR971**	Clinical isolate from a 9 year-old child with sinusitis	this study

We have previously shown that MID binds both secreted and membrane bound IgD [11],[26]. The human serum concentration of IgD is very low [27], *i.e*., IgD represents less than 0.25% of the total Ig concentration in serum. To determine whether IgD could block the IgD-dependent bacterial interaction with B cells, FITC-labelled *M*. *catarrhalis* was incubated with tonsillar B cells in the presence of purified serum IgD. At the highest physiological IgD concentration tested (50 µg/ml), no decrease in bacterial binding to B cells was detected as compared to the control without IgD (Fig. 1G). However, when the IgD concentration was increased up to three-fold (150 µg/ml), a 40% reduction of binding to B cells was found. To exclude that IgD-binding to FITC-*Moraxella* was not quenched by the FITC conjugation, the capacity of FITC-labelled *M*. *catarrhalis* to bind purified IgD was also tested. A preserved bacterial IgD-binding was found when IgD binding was analysed with an RPE-conjugated rabbit anti-human IgD pAb (Fig. 1G; inserts). Our findings raised the question why *Moraxella* is equipped with the superantigen MID, since expression of MID would be potentially harmful for bacterial survival when endocytosed by B cells.

### *Moraxella* OMV bind to B lymphocytes by a MID-dependent mechanism

**Figure 2:**
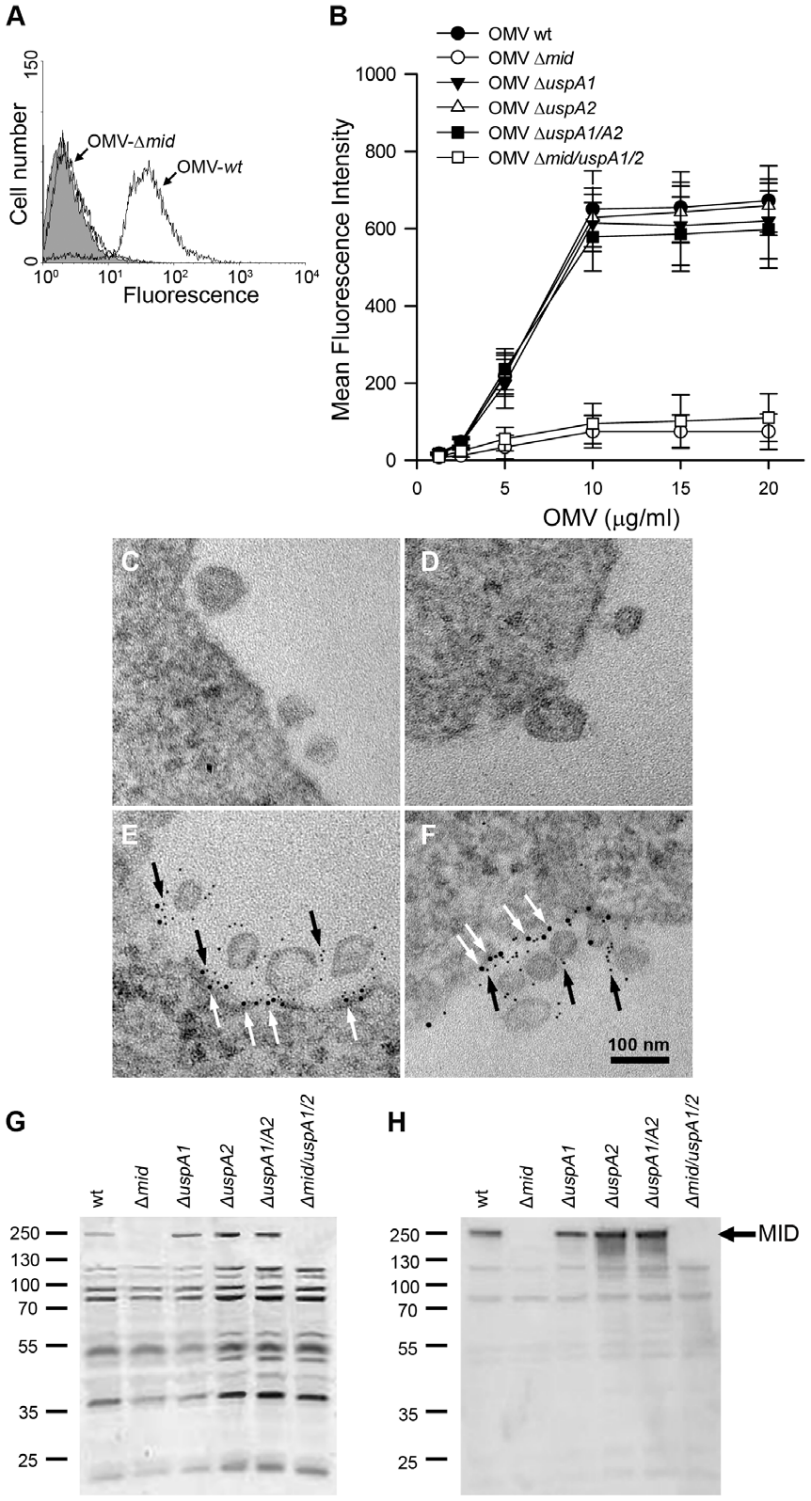
*M*. *catarrhalis* OMV bind to tonsillar lymphocytes through an interaction via MID and the IgD B cell receptor. (A) A typical flow cytometry experiment with OMV and B cells. Control B lymphocytes (shaded) and cells incubated with FITC-labelled OMV wild type (OMV-wt) or MID-deficient OMV (OMV-Δ*mid*). OMV were used at 10 µg/ml and incubated with B cells for 1 h at 37°C. (B) Concentration-dependent binding of OMV to B cells. Increased concentrations of FITC-labeled OMV were incubated with B cells for 1 h followed by flow cytometry analyses. Data are presented as mean±SEM of 3 experiments. (C, D) B cells and OMV interactions analyzed in TEM showed binding of several OMV to the B cell surface. (E, F) IgD molecules were clustered upon OMV binding: gold-labelled antibodies demonstrated formation of a tight cluster of IgD (large granules; white arrows) and MID (small granules; black arrows). The bars represent 100 nm in length. The expression of MID in OMV isolated from different *M*. *catarrhalis* isogenic mutants was analyzed by SDS-PAGE (G) and Western blot (H) using MID specific rabbit pAbs. Molecular weight markers in kilodalton are indicated to the left.

OMV secreted by *M*. *catarrhalis* would theoretically also contain MID as vesicles basically comprise outer membrane components, i.e., LPS, phospholipids and outer membrane proteins. The potential of OMV to carry and long-distance deliver diverse virulence factors prompted us to hypothesize that *Moraxella* OMV can activate human B cells as has been proven with whole bacteria [13],[14]. The initial step in B cell activation requires *M*. *catarrhalis* OMV binding to B cells. To study the capacity of OMV to interact with tonsillar B cells, OMV isolated from overnight cultures of different *M*. *catarrhalis* strains were labelled with FITC and incubated with purified B cells for 1 h. After several washing steps, the binding of FITC-conjugated OMV was analyzed by flow cytometry (Fig. 2A). The binding of OMV to B cells was concentration dependent and saturated above 10 µg/ml (Fig. 2B). In agreement with our previous observations based on analysis of whole bacteria [13], the interaction between B cells and OMV involved the presence of the IgD-binding outer membrane protein MID as OMV isolated from MID-deficient mutants (OMV Δ*mid*), barely bound to purified B cells at the different concentrations tested (Fig. 2A and B). Moreover, OMV isolated from mutants deficient in ubiquitous surface protein (Usp) A1 and A2, two other important multifunctional *Moraxella* surface virulence factors [28],[29], were also shown to bind to tonsillar B cells at the same level as the wild type MID-containing OMV.

The interaction between OMV and B cells was further analyzed by transmission electron microscopy (TEM) after co-culturing B cells with live *M*. *catarrhalis* for 1 h allowing them to produce natural OMV. The *M*. *catarrhalis* OMV clearly bound to the B cell surface as can be seen in Fig. 2C and D. In contrast and in parallel to our flow cytometry experiments, MID deficient OMV did not significantly bind to B cells as revealed by TEM (data not shown). The interplay between MID-containing OMV and the IgD B cell receptor (BCR) on host cells was also observed using gold-labelled antibodies directed against either IgD (large granules; white arrows) or the MID protein (small granules; black arrows) (Fig. 2E and F). We found that on the average 83% of the large gold granules (representing the IgD BCR) and 79% of the small gold particles (MID) were associated with plasma membranes and OMV, respectively. In addition, 36% of all gold particles were found colocalized at the interface between OMV and the plasma membrane.

To confirm the presence or absence of MID in the different OMV preparations, OMV were analyzed by SDS-PAGE and Western blot using rabbit pAbs directed against the KTRASS repeat within the IgD binding region of MID [10] (Fig. 2G and H). Thus, OMV originating from the MID-expressing *M*. *catarrhalis* BBH18 wild type (wt) and UspA-deficient mutants contained the outer membrane protein MID, whereas OMV obtained from the MID-deficient mutants *M*. *catarrhalis* were consequently deficient in MID.

### OMV induce Ca^2+^ mobilization and clustering of B cell receptors by lipid rafts

**Figure 3:**
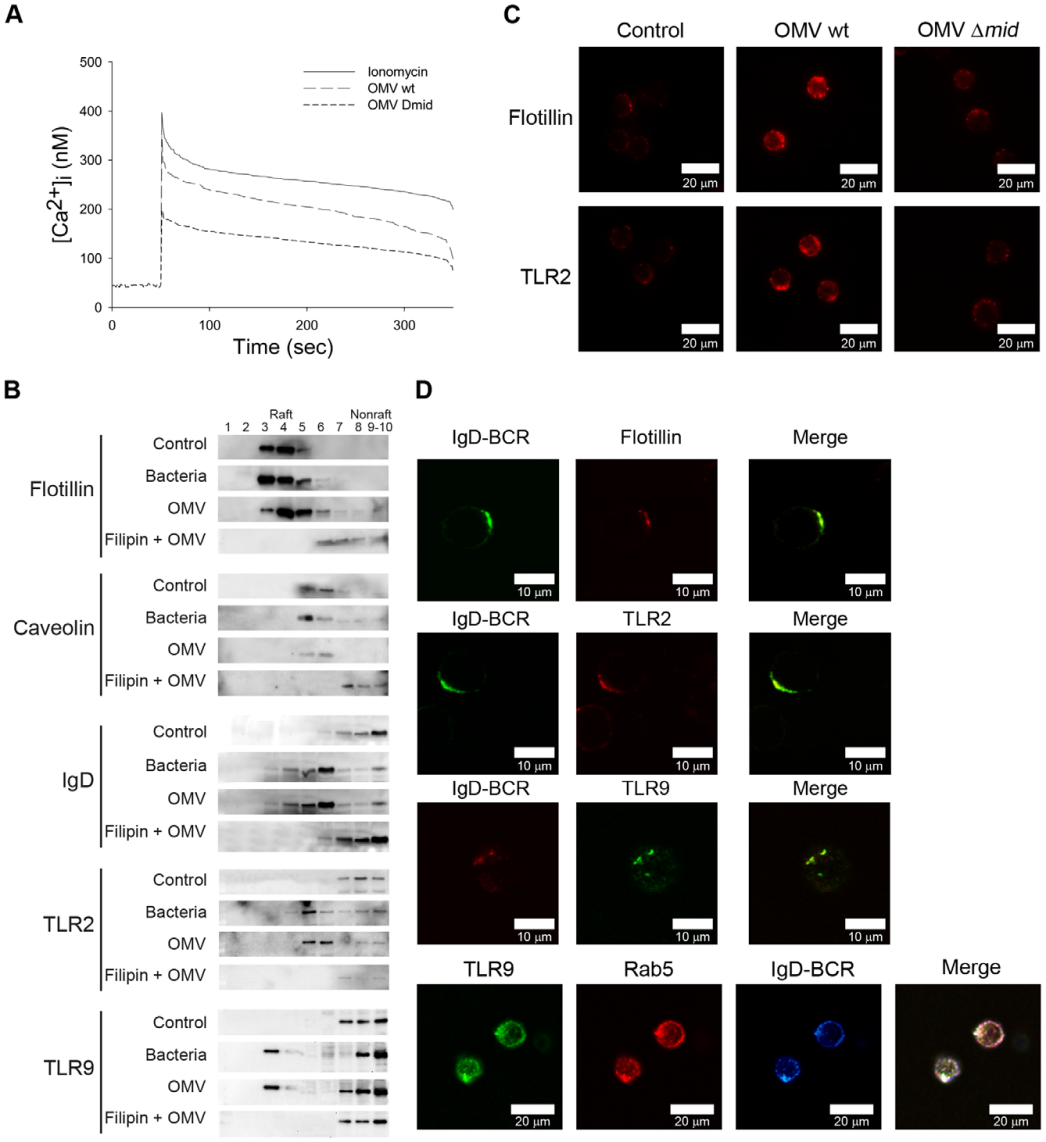
Ca^2+^ mobilization and B cell receptor clustering are induced by OMV. (A) Mobilization of [Ca^2+^]_i_ in human tonsillar B cells induced by *Moraxella* OMV. Purified Fura-2 loaded B cells were stimulated with wild-type OMV (10 µg/ml), MID-deficient OMV (10 µg/ml) or ionomycin (100 nM). Ca^2+^ mobilization was analyzed as described in Materials and Methods. Each experiment was repeated with at least three different donors with comparable results. (B) Aliquots of Triton-insoluble lysates of B cells obtained before (Control) and after stimulation with formaldehyde-fixed *M*. *catarrhalis* wild type (Bacteria), OMV or treated with filipin prior to stimulation with OMV (Filipin + OMV) were fractionated on discontinuous sucrose gradients and were immunoblotted with anti-flotillin, anti-caveolin, anti-TLR2, anti-IgD or anti-TLR9 mAbs. (C) Visualization of receptor clustering in lipid rafts. B cells unstimulated (Control), wild type OMV (OMV wt) or MID-deficient OMV (OMV Δ*mid*) stimulated for 30 min were fixed and stained with anti-flotillin (top) or anti-TLR2 (bottom). Alexa Fluor 594 goat anti-mouse IgG pAb were used as a secondary layer. (D) Colocalization of flotillin, TLR2, Rab5 and TLR9 with IgD (BCR) confirmed by confocal microscopy. Purified B cells were incubated with OMV for 30 min, fixed and stained with FITC-conjugated or RPE-conjugated mAbs against IgD (BCR) and FITC-conjugated anti-TLR9 mAb, anti-flotillin mAb or anti-TLR2 mAb using Alexa Fluor 594-conjugated secondary pAb. For the triple staining, stimulated B cells were incubated with rabbit anti-IgD pAb (BCR) and mouse anti-Rab5 mAb (Rab5). After several washes, B cells were incubated with Alexa Fluor 633 goat anti-mouse IgG and Alexa Fluor 546 goat anti-rabbit IgG secondary mAb followed by incubation with FITC-conjugated anti-TLR9 mAb (TLR9). The lymphocytes shown are representatives of more than 90% of the cells imaged in 3 separate experiments with different donors.

BCR cross-linking results in the recruitment and activation of several signaling components and triggers the generation of second messengers, *i.e*., inositol 1,4,5-triphosphate (InsP3) and diacylglycerol. InsP3 induces calcium release from internal stores into the cytoplasm and promotes calcium entrance through the plasma membrane which ultimately increases the intracellular calcium concentration and results in activation of gene expression. To determine whether the binding of OMV mobilizes Ca^2+^ ions in human tonsillar B lymphocytes, we measured Ca^2+^ mobilization in Fura-2 loaded cells. Ionomycin was used as a positive control, and as shown in Figure 3A, OMV at 10 µg/ml induced a rapid, strong and transient elevation of [Ca^2+^]_i_ in B cells. MID-deficient OMV also induced a Ca^2+^ mobilization although significantly lower as compared to the wild type OMV.

Ligation of the BCR with antigen induces lipid raft coalescence, a process that allows concentration of key signaling molecules and promotes contact between them to ensure efficient and sustained signal transduction [30]–[32]. In initial experiments our TEM analysis revealed a clustering of the IgD BCR at the B cell surface when cells were exposed to MID-containing OMV (Fig. 2F; white arrows). Biochemical evidence of lipid rafts in B cells was obtained by the isolation of Triton-insoluble material from purified B cells before and after exposure to OMV or formaldehyde-fixed *M*. *catarrhalis* wild type that were used as a positive control. The Triton-insoluble material was then fractionated on a discontinuous sucrose gradient and aliquots were screened by Western blots with specific antibodies (Fig. 3B). Flotillin and caveolin were used as markers for raft fractions [33]. Importantly, IgD, TLR2 and TLR9 were found in the raft fractions of cells stimulated with either bacteria or OMV. To confirm the compartmentalization of receptors into lipid rafts, we treated B cells with filipin, which intercalates into lipid motifs and hereby disrupts lipid raft structures [34]. The partitioning of TLR2, TLR9 and IgD induced by OMV was prevented in B cells treated with filipin and thus the lipid raft fractions did not include these molecules (Fig. 3B).

Fluorescence and confocal microscopy also revealed that lipid raft domains coalesce into large patches on the B cell surface after OMV stimulation. Unstimulated B cells exhibited a weak generalized membrane flotillin and TLR2 staining, which we assume indicated the presence of small lipid rafts. BCR stimulation via MID-containing OMV (OMV wt) caused a clear polarization of lipid rafts as was observed by the punctuate staining over a few regions of each cell (Fig. 3C). The percentage of B cells stimulated with OMV wt showing polarization of lipid rafts was 89.7±2% (mean value±SD, *n* = 7 different experiments) compared to 1.1±1% of B cells stimulated with MID-deficient OMV. Colocalization between Flotillin, TLR2 or TLR9 and IgD BCR induced by OMV was also confirmed by double staining of stimulated B cells followed by confocal analysis (Fig. 3D). Interestingly, IgD BCR and TLR9 colocalized with the early endosomal marker Rab5 on B cell endosomal compartments after OMV stimulation for 30 min. Thus, by using both TEM (Fig. 2F) and biochemical data in addition to confocal microscopy we have shown that OMV binding initiates IgD BCR clustering.

### OMV are taken up by human B cells through a BCR-mediated mechanism

**Figure 4:**
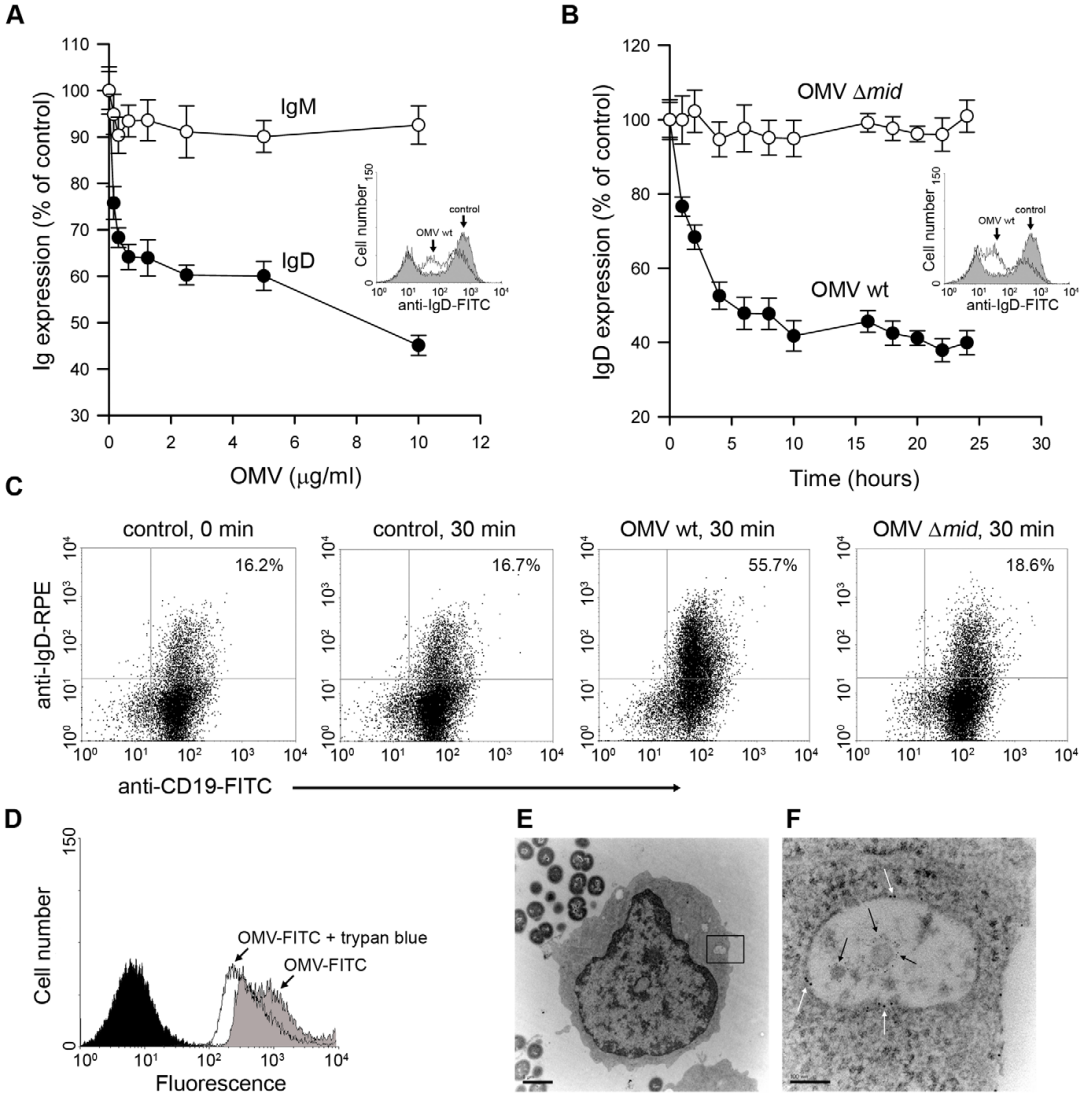
OMV induce a down-regulation of surface expressed IgD in a dose dependent manner and are internalised by tonsilar B cells. Changes in surface expression of IgD and IgM after OMV binding to B cells were analyzed by flow cytometry. (A) Purified tonsil B cells were incubated with different concentrations of OMV ranging from 150 ng/ml to 10 µg/ml and monitored for IgD and IgM expression after 24 h at 37°C. Insert shows a representative flow cytometry profile of IgD-labelled B cells that were left unstimulated (shaded) or stimulated with OMV (white). (B) The kinetics of IgD internalization was examined after incubation with 10 µg of either OMV wild type (OMV-wt) or MID-deficient OMV (OMV-Δ*mid*). Data are shown as arbitrary units comparing Ig densities at different time-points with expression at time 0 h set to 100%. Error bars indicate SEM from four and three different donors in panels A and B, respectively. Insert shows a representative flow cytometry profile of IgD-labelled B cells that were unstimulated (shaded) or stimulated with OMV wt (white). (C) The IgD internalization after OMV stimulation was examined by flow cytometry. Unstimulated (control) B cells or B cells stimulated with 10 µg/ml of OMV wild type (OMV wt) or MID-deficient OMV (OMV Δ*mid*) were fixed and incubated with FITC-conjugated anti-CD19 mAb and rabbit anti-IgD pAb to block the surface expressed BCR. After several washes, B cells were permeabilized and incubated with RPE-conjugated anti-IgD mAb and analyzed by flow cytometry. The data shown are representative of those from three independent experiments. (D) Representative flow cytometry experiment of OMV entry into B cells. Control B cells (shaded) and cells incubated with FITC–labeled OMV before (OMV-FITC) and after quenching with trypan blue (OMV-FITC + trypan blue). (E, F) B cells and OMV interactions analyzed in TEM showed internalisation of MID containing OMV. The endosome with two OMV is magnified in (F) and gold-labelled antibodies against IgD (large granules; white arrows) and MID (small granules; black arrows) are indicated. The scale in (E) and (F) represents 1 µm and 100 nm in length, respectively.

Antigen-induced clustering of the BCR is normally followed by BCR internalization and movement to endosomal compartments. To explore the expression of IgD after binding of OMV, B cells were incubated in the presence of different OMV concentrations. Cells were harvested after 24 h and analyzed for changes in IgD and IgM expression using flow cytometry analysis. The surface expressed IgD was strongly down regulated in the presence of OMV (Fig. 4A). In contrast, IgM density was not affected by OMV. The kinetics of the OMV-induced IgD down-regulation was further analyzed comparing IgD expression on B cells incubated with either wild type OMV or MID-deficient OMV (Fig. 4B). A significant decrease in IgD density was detected as early as after one hour of incubation with MID-containing OMV and reached the lowest value after 10 h. As expected, the IgD expression was not affected in the presence of MID-deficient OMV. To relatively quantify the internalization of the IgD BCR after OMV stimulation, intracellular IgD was analysed in tonsillar B cells stimulated for 30 min with either MID-containing or MID-deficient OMV (Fig. 4C). The number of CD19^+^ lymphocytes with intracellular IgD increased 28.1±9% (mean value±SD, *n* = 4) as compared to unstimulated B cells or cells stimulated with MID-deficient OMV.

To further study the entry of OMV into B cells, we used a fluorescence quenching method [35]. FITC-labeled OMV were incubated with B cells for 1 h, followed by extensive washing steps and flow cytometry analysis. The mean fluorescence of cells with bound OMV (extra- and intracellular) was compared with intracellular OMV after addition of trypan blue, which quenches the extracellular FITC signal (*i.e*., only intracellular OMV would then be fluorescent). As shown in Figure 4D, after 1 h incubation with FITC-conjugated OMV 76.6±2.3% of the B cells had internalized the OMV-FITC. Notably, two populations of B cells with high and low fluorescence were seen corresponding to intracellular and extracellular OMV-FITC. To confirm the OMV internalization by TEM, purified B cells were incubated with *M*. *catarrhalis* for 1 h to allow the secretion of OMV. Figure 4E and 4F show a B cell that has internalized at least two MID-containing OMV in an endosome. Interestingly, the endosomes were coated with IgD (large granules; white arrows). MID was also visualized with a gold-labelled pAb (small granules; black arrows). As expected, MID-deficient OMV were not taken up by the B lymphocytes (results not shown). Taken together, the interaction between MID and IgD induced OMV internalization by a receptor mediated endocytosis.

### *M*. *catarrhalis* OMV activate human tonsillar B cells

**Figure 5:**
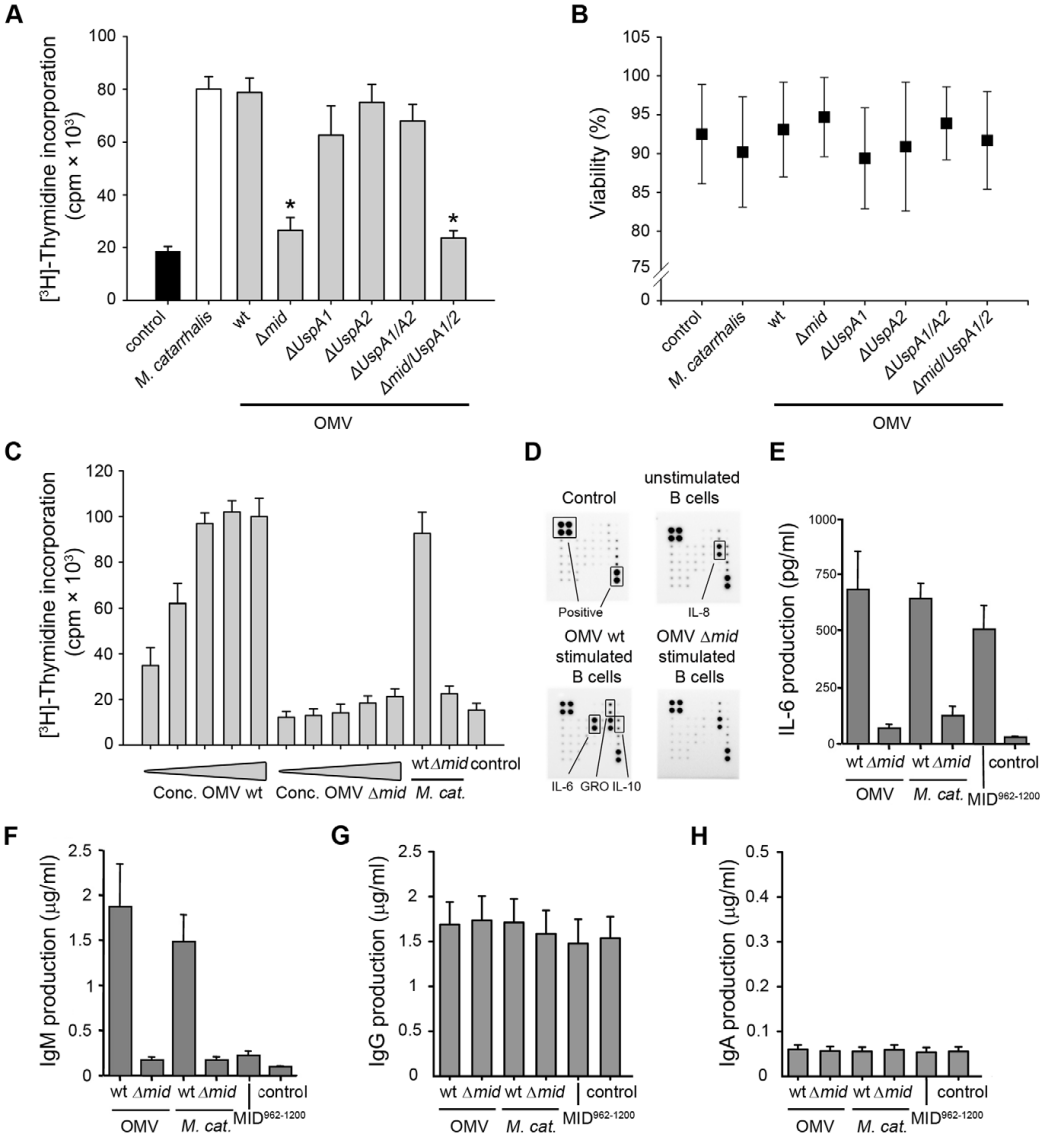
OMV containing MID activate tonsillar B cells. (A) The mitogenic effect of *M*. *catarrhalis* OMV isolated from different strains on B cells was analyzed by measuring thymidine uptake at 96 h. Error bars indicating SEM from 6 different donors, * p≤0.05, OMV versus control. (B) The viability of B lymphocytes after OMV stimulation was assessed by trypan blue exclusion staining. (C) Purified B cells were stimulated with different concentrations of OMV with or without MID (OMV Δ*mid*), whole *M*. *catarrhalis* wild type or its isogenic mutant or recombinant MID^962-1200^ as indicated. The OMV concentrations used were in the range of 0.1–25 µg/ml. Error bars indicate SEM from 5 different donors. (D) OMV activated B cells mainly produce IL-6. Purified B cells were incubated with 10 µg/ml OMV wild type (OMV wt) or MID-deficient OMV (OMV Δ*mid*) for 96 h. Thereafter, the cytokine contents in the B cell culture supernatants were determined using a human cytokine protein array. The protein microarray cut-off controls are indicated as positive controls. Complete medium and a culture supernatant from unstimulated B cells were included as a negative control. A minor up-regulation of IL-10 and GRO was also detected. (E–H) OMV binding to B cells induces IL-6 and IgM secretion. The stimulatory effect of *M*. *catarrhalis* OMV on B cells was analyzed by measuring IL-6 secretion at 48 h (E) and IgM (F), IgG (G) or IgA (H) production at 96 h. B cells were activated with 10 µg/ml OMV isolated from MID-expressing bacteria (wt) or a MID-deficient mutant (*Δmid*). In addition, whole *M*. *catarrhalis* wild type (wt), its isogenic mutant (*Δmid*), or recombinant MID^962-1200^ were included in the analysis. Error bars indicate SEM from 5 (E) and 3 (F–H) different donors.

To evaluate the capacity of *Moraxella* OMV to activate human B lymphocytes, freshly isolated tonsillar B cells were incubated with OMV isolated from different strains of *M*. *catarrhalis* and analyzed for [methyl-^3^H]-thymidine incorporation after 96 h, using whole bacteria killed by formaldehyde as a positive control [12]. In contrast to OMV from MID-deficient mutants, only OMV isolated from MID-expressing strains were found to have a similar B cell stimulatory effect as compared to whole *M*. *catarrhalis* (Fig. 5A). Any changes in B cell viability was not observed after stimulation with the different *Moraxella* OMV preparations as compared to B cells incubated with whole bacteria (Fig. 5B). OMV isolated from the UspA1- and A2 deficient isogenic (but MID expressing) control mutants did not interfere with B cell function. The mitogenic effect of OMV containing the MID protein was concentration dependent and saturated above 1.5 µg/ml (Fig. 5C).

To characterize the OMV-dependent B cell activation in detail, supernatants from B cell cultures stimulated with OMV from the *M*. *catarrhalis* wild type (wt) or the MID-deficient mutant were analyzed using a human cytokine array containing antibodies directed against different chemokines, interleukins (IL) and growth factors, including IL-6, IL-10, tumor necrosis factor (TNF)-α, and TNF-β. A strongly increased IL-6 production, up to 55-fold, was detected when B cells were activated with the MID-containing wild type OMV as compared to the MID-deficient OMV (Fig. 5D). IL-8 secretion was found irrespectively of incubation with OMV as compared to complete medium that was included as a negative control. In addition, a minor up-regulation of IL-10 (3-fold increase) and the chemokine growth regulated oncogene (GRO) (2-fold increase) was also observed in cell cultures with OMV-stimulated B lymphocytes.

To more precisely quantify the IL-6 concentrations in cultures with OMV-stimulated B cells and compare the IL-6 response in cultures with B cells activated in the presence of whole bacteria, an ELISA was included in our analysis. Cell free supernatants from different time points (24 to 96 h) were analyzed. The IL-6 synthesis reached a maximum after 48 h and did not change at later time points (data not shown). As exemplified in Figure 5E, OMV induced a strong IL-6 response at 48 h. MID-expressing whole bacteria or the IgD-binding recombinant MID^962-1200^ fragment [8],[10] coated to the bottom of wells induced a similar response suggesting that IgD cross-linking was the main factor for IL-6 production. In parallel, IL-6 concentrations in B cell cultures incubated with MID-deficient OMV or the mutant *M*. *catarrhalis* Δ*mid* were comparable with background values.

To determine whether OMV could induce Ig synthesis in tonsillar B lymphocytes, supernatants from purified B cells stimulated with *Moraxella* OMV were analyzed for Ig content by ELISA (Fig. 5F–H). Cell free supernatants from B cells stimulated with whole bacteria or recombinant MID^962-1200^ were analyzed in parallel as controls. Wild type OMV induced higher levels of IgM production as compared to MID-deficient OMV (Fig. 5F), whereas no differences were detected regarding IgG and IgA synthesis (Fig. 5G and H). This indicated that the stimulation induced by OMV was not enough to drive class switch recombination (CSR). In parallel to the results on IL-6 (Fig. 5E), only small variations in IgM production were detected between MID-expressing whole *M*. *catarrhalis* bacteria and its derived OMV (Fig. 5F). However, any secreted IgM was not detected in the other supernatants including B cells activated with the recombinant MID^962-1200^ supporting previous observations that highlighted the need for additional PRR signals or T cell-mediated help (CD40L or cytokines) for optimal B cell activation [12]–[14].

The specificity of the OMV-induced antibodies was also tested using a panel of the most dominant respiratory pathogens (*M*. *catarrhalis*, non-typeable *H*. *influenzae* (NTHi), encapsulated *H*. *influenzae* serotype b (Hib), and *S*. *pneumoniae*). Bacteria were incubated with cell-free supernatants from OMV-stimulated B cells and screened by flow cytometry for binding of IgM (Table 2) and IgG (not shown). In agreement with our previous results with whole *Moraxella* bacteria [14], no differences were detected between specific antibody binding and background values to any of the screened pathogens. These results suggested that OMV-induced antibody production may not help the host to clear *Moraxella* but merely redirects the humoral response.

**Table 2:** Analysis of the specificity of IgM in supernatants from human B cells stimulated with OMV.

	IgM^a^ in B cell supernatant				
Species and strain n°	unstimulated	OMV wt stimulated	OMV Δ*mid* stimulated	Control^b^	H serum^c^
*S*. *pneumoniae* ATCC 6303	20.0±1.9	19.9±2.6	18.7±2.2	20.2±2.0	64.7±4.5
*S*. *pneumoniae* S6-2489	26.7±4.1	26.3±3.3	26.1±4.4	25.7±1.7	28.4±3.9
*H*. *influenzae* type b 41	17.6±3.1	19.0±4.0	17.6±3.7	20.9±3.5	30.7±2.4
*H*. *influenzae* type b 43	17.2±1.7	17.7±2.4	17.8±2.6	18.3±2.7	19.5±1.6
non-typeable *H*. *influenzae* 722	20.4±1.2	17.8±2.1	17.6±1.9	18.4±1.5	18.7±2.3
non-typeable *H*. *influenzae* 541	17.5±3.4	17.3±5.1	16.4±4.7	24.3±3.4	60.1±5.3
*M. catarrhalis* BBH18	26.2±1.7	22.9±2.5	24.6±3.1	27.3±2.8	36.8±4.2
*M*. *catarrhalis* KR 923	24.6±2.1	23.9±1.6	25.6±1.9	26.6±3.6	42.6±5.2

a Average values of mean fluorescence intensity±SEM from three independent experiments with separate tonsil donors. Bacteria were incubated with supernatants from B cell cultures as indicated followed by incubation with FITC-conjugated rabbit anti-human IgM and flow cytometry analyses.

b The negative control consisted of complete culture medium (RPMI 1640) without human serum and was used as a control for unspecific binding of the FITC-conjugated rabbit anti-human IgM.

c Complete culture medium (RPMI 1640) supplemented with a pool of human sera to a final dilution of 1/1,000 was used as a positive control.

**Figure 6:**
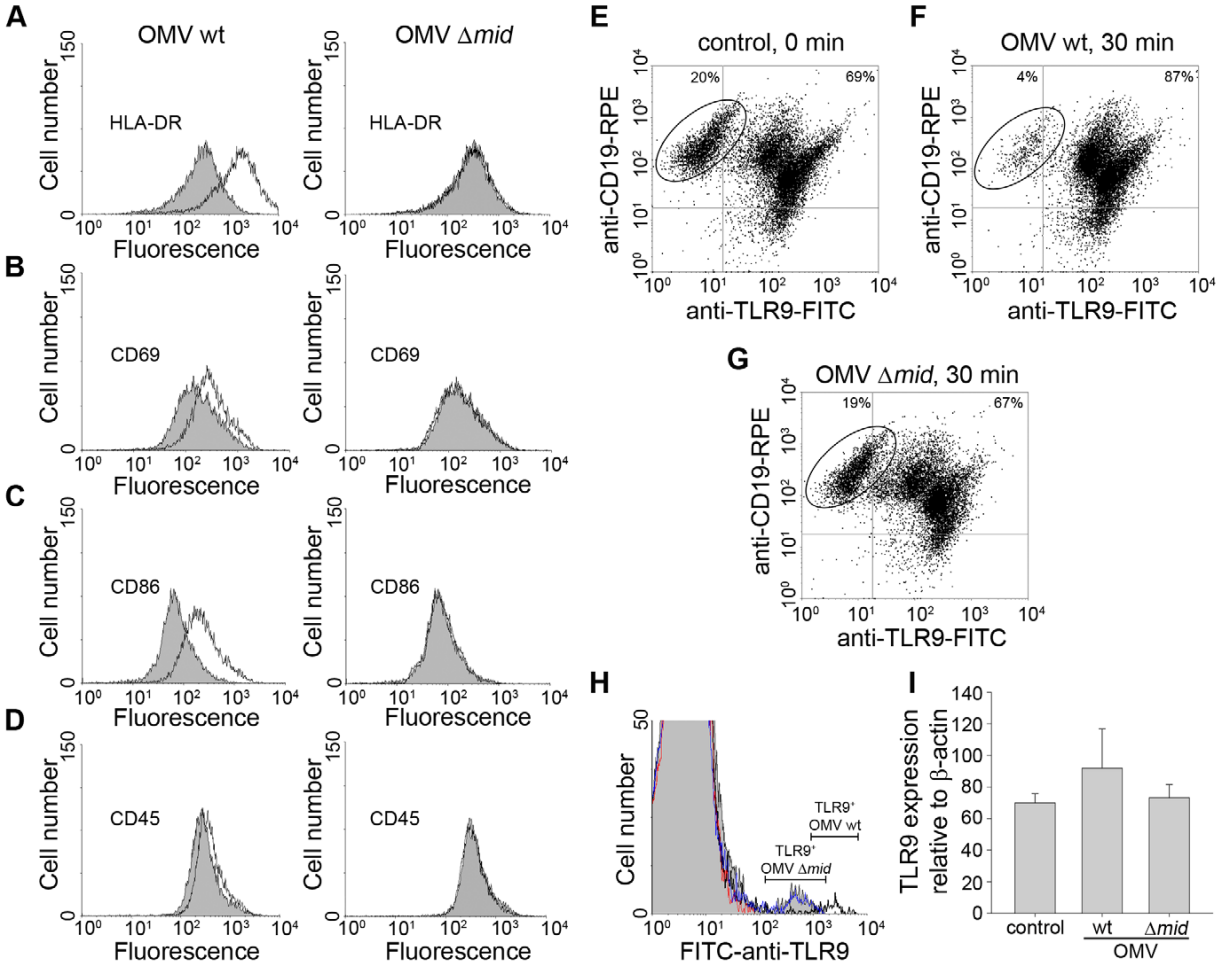
OMV cause up-regulation of B cell surface markers. OMV induced changes in expression of HLA-DR (A), CD86 (B), CD69 (C) and CD45 (D). Surface expression of B cell activation markers after OMV stimulation was analyzed by flow cytometry. Unstimulated B cells are shown as filled profiles (gray) and B cells incubated with OMV wt (left panels) or MID-deficient OMV (OMV Δ*mid*, right panels) are indicated as white profiles. Representative results from one out of three donors are shown. (E–G) Flow cytometry analysis of TLR9 expression in B cells stimulated with OMV. Purified B cells were either unstimulated (E) or incubated with 10 µg/ml of OMV-wt (F) or OMV-Δ*mid* (G) for 30 min. Thereafter, B cells were double stained with RPE-conjugated anti-CD19 and FITC-conjugated anti-TLR9 mAbs followed by flow cytometry analysis. In panels (E) to (G), the TLR9-negative CD19^+^ B cell population is encircled to illustrate the CD19^+^TLR9^−^ population that decreased after stimulation. Data are representative for 3 similar profiles obtained from separate donors. (H) Flow cytometry analysis of surface TLR9 expression in OMV-stimulated B cells. Unstimulated B cells are shown as a grey profile. B cells stimulated with OMV wt or MID-deficient OMV for 30 min are shown as dark black and blue line profile, respectively. Isotype control Ab-labelled B cells are shown as a red line profile. One representative histogram is shown out of three independent experiments. (I) Relative levels of TLR9 transcripts in OMV-stimulated human tonsillar B cells. Purified B cells were either unstimulated (control) or incubated with 10 µg/ml OMV wild type (OMV wt) or MID-deficient OMV (OMV Δ*mid*) for 30 min. Expression levels were determined using quantitative real-time reverse transcription-polymerase chain reaction (RT-PCR) and are depicted in relation to the internal control gene, β-actin, as 2^ΔCt^×10^5^. Data from experiments with cells from 3 different donors are summarized and presented as mean±SEM.

A triggered BCR initiates a signaling cascade that leads to up-regulation of co-stimulatory molecules. To examine the effect of OMV on B cell surface molecules, purified tonsillar B cells were incubated with OMV and screened by flow cytometry for HLA-DR, CD69, CD86, and CD45 surface expression. As seen in Figure 6A–D, only MID-containing OMV induced up-regulation of these antigens and receptors. Thus, the B cell phenotype induced by OMV was similar to the one observed in B cells stimulated with whole *M*. *catarrhalis* [14].

B cell expression of certain TLRs is important in linking innate and adaptive immune responses. Several studies have shown that TLR9 is highly expressed in B cells [36],[37]. It has recently been demonstrated that peripheral blood B lymphocytes (CD19^+^) can be separated into two subsets consisting of TLR9^−^ and TLR9^+^ populations [38]. To study whether TLR9 expression was affected by OMV, tonsillar B cells were examined by flow cytometry analyses after OMV exposure. Stimulated lymphocytes were permeabilized and double stained using RPE-conjugated anti-CD19 and FITC-conjugated anti-TLR9 mAbs. Both TLR9^−^ and TLR9^+^ subpopulations were found in the human tonsil B cell compartment (Fig. 6E). Moreover, we were able to detect two subpopulations; low and high TLR9 expressing CD19^+^ B lymphocytes, which most likely illustrated the presence of different stages of B cell differentiation in the tonsillar tissue. After 30 min of incubation, however, 87±2% of the CD19^+^ cells incubated with MID-containing OMV were TLR9^+^ (Fig. 6F) as compared to 67±3% of CD19^+^ B cells in cultures exposed to OMV isolated from the isogenic mutant *M*. *catarrhalis* Δ*mid* (Fig. 6G). The CD19^+^TLR9^−^ population that decreased in density after activation is encircled in Figure 6E to G.

Intriguingly, it has been demonstrated that TLR9 can be expressed at the cell surface of a subpopulation of tonsillar B cells [39]. In that particular study, B cells positive for surface TLR9 expression represented a minor fraction of the total B cell population, varying between 2 and 10%. We also detected a minor surface TLR9^+^ subset of tonsillar B cells (Fig. 6H). The percentage of surface TLR9^+^ B cells was 5.4±1.4 (mean value±SD, *n* = 3) of the total B lymphocytes relative to the isotype control (0.4±0.9%). Lymphocytes stimulated for 30 min with MID-containing OMV showed a two-fold higher cell surface level of TLR9 (mean fluorescence intensity [mfi]: 715±54 [mean value±SD, *n* = 3) than did MID-deficient OMV stimulated cells (mfi, 360±65) (Fig. 6H). To further analyse this phenomenon, TLR9 transcripts levels in relation to the housekeeping gene β-actin were measured using a quantitative real-time PCR. However, despite an apparently higher level of TLR9 transcripts observed in B cells stimulated with OMV as compared with B cells activated with MID-deficient OMV, the difference was not statistically significant (Fig. 6I). To summarize, in addition to a change in the B cell surface phenotype upon stimulation with OMV, the expression of the DNA receptor TLR9 increased.

### OMV activate B cells via IgD BCR cross-linking and TLR9 signaling

**Figure 7:**
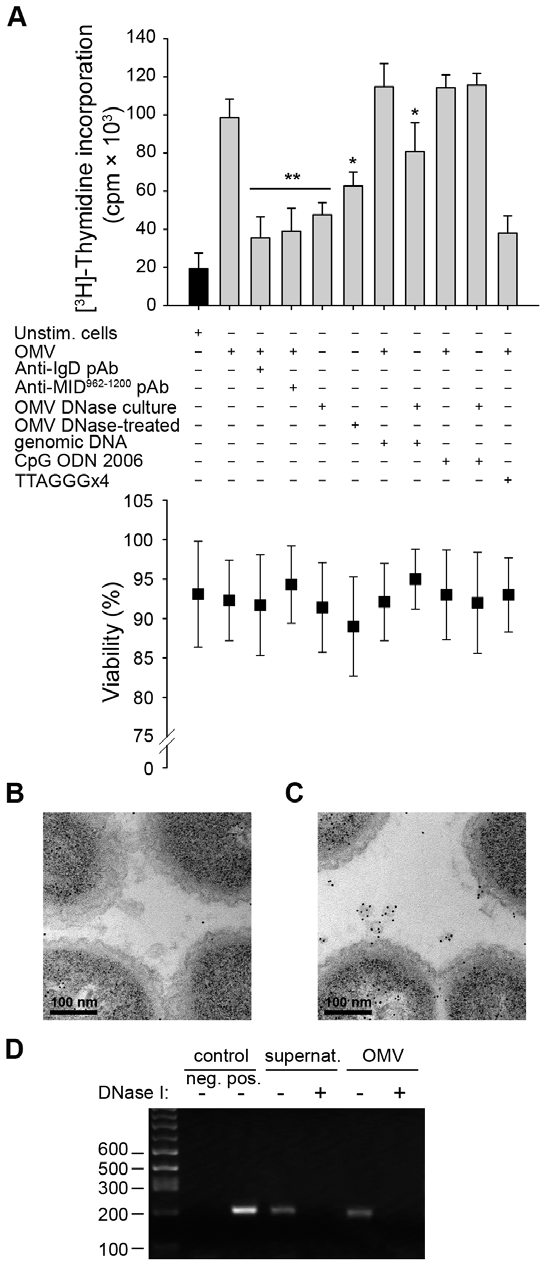
TLR9 participates in OMV-dependent B cell activation. (A) B cell proliferation was measured by means of [methyl-^3^H]-thymidine incorporation after 96 h. OMV from the MID-expressing *M*. *catarrhalis* wild type (OMV) were combined with different additives as indicated in the figure. OMV isolated from *M*. *catarrhalis* growing in presence of DNase (OMV DNase culture) and OMV treated with DNase after isolation (OMV DNase-treated) were also included. The viability of B lymphocytes after each treatment was measured by trypan blue exclusion staining. Error bars indicate SEM from 5 different donors. The presence of DNA associated with OMV from DNase treated bacterial culture (B) or untreated culture (C) were analyzed by TEM using gold labelling anti-DNA pAb. (D) The presence of DNA associated with OMV was confirmed by PCR. DNA extractions from OMV DNase-treated or untreated samples were used as template for amplification of the genomic 16S rRNA gene. *M*. *catarrhalis* genomic DNA was used as a positive control and an ultracentrifuge supernatant (free of bacteria and OMV) from DNase treated and untreated cultures were also analyzed to check the presence of extracellular DNA. Representative images from three independent experiments are shown. * p≤0.05, OMV DNase-treated versus OMV and OMV DNase-treated + genomic DNA versus OMV DNase-treated; ** p≤0.01, OMV versus unstimulated cells.

The natural ligand for TLR9 is unmethylated CpG-DNA motifs, which are primarily found in viral and bacterial DNA. Previous studies demonstrated that OMV secreted by pathogenic bacteria carry luminal DNA as well as DNA on their surface [21],[40],[41]. To further investigate the role of TLR9 in *Moraxella* OMV-induced B cell activation, we incubated human tonsillar B cells with OMV isolated from bacteria grown in the presence of DNase (“OMV DNase culture”) or OMV treated with DNase after isolation (“OMV DNase-treated”) (Fig. 7A). Interestingly, a synergistic effect was seen upon stimulation with a combination of IgD (via OMV) and TLR9 (via genomic *Moraxella* DNA or the synthetic TLR9 ligand CpG ODN 2006). The B cell proliferation was significantly reduced with OMV isolated from bacteria that had been cultured in the presence of DNase (Fig. 7A). Furthermore, B cell stimulation induced by OMV from DNase-treated cultures was similar to the activation detected by preincubation of B cells with antibodies directed against either the IgD BCR or MID, which also inhibited the OMV-dependent B cell activation by neutralizing the IgD cross-linking. Importantly, the presence of genomic *Moraxella* DNA or CpG ODN 2006 restored the proliferation induced by OMV isolated from DNase-treated cultures and was comparable to the activation induced by DNA-containing OMV. Finally, the B cell proliferation induced by OMV was significantly reduced in the presence of the dominant negative TLR9 inhibitory oligonucleotide (TTAGGG)×4 (Fig. 7A).

The absence of DNA in OMV preparations from bacteria growing in the presence of DNase was further confirmed by gold-conjugated antibodies directed against DNA using TEM (Fig. 7B), and by PCR amplification of the 16S rRNA gene of *M*. *catarrhalis* (Fig. 7D). In contrast, DNA associated with OMV secreted by untreated (*i.e.,* no DNase supplemented) *Moraxella* cultures was observed (Fig. 7C and D). Quantification of labelled DNA in the TEM images revealed 39.4 gold particles/square micrometer in untreated *Moraxella* cultures (Fig. 7C) as compared to 4.7 gold particles/square micrometer in the DNase-treated cultures (Fig. 7B). In conclusion, *Moraxella* OMV induced a B cell signal via the IgD BCR, whereas the second signal was mediated via TLR9 and DNA containing CpG-motifs associated with OMV.

### Secretion of MID-containing OMV by *M*. *catarrhalis in vivo*

**Figure 8:**
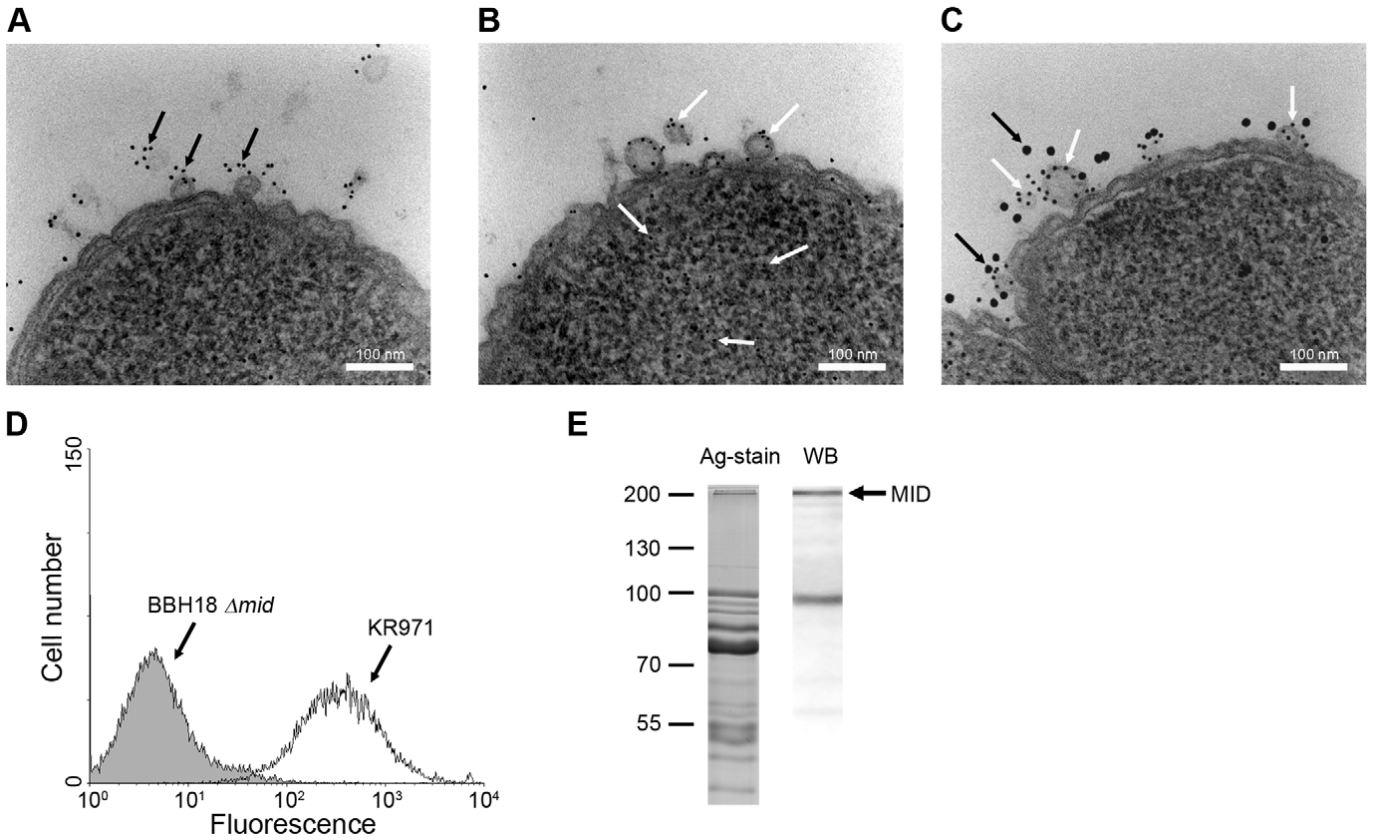
*M*. *catarrhalis* OMV secreted *in vivo* contain MID and DNA. A clinical sample from a 9-year old patient was analysed and TEM showed the presence of MID (A) and DNA (B) on OMV. In (C) MID and DNA associated with OMV is marked with gold-labelled antibodies against MID (large granules, black arrows) and DNA (small granules, white arrows). The scale bar in (A–C) represents 100 nm in length. (D) Representative flow cytometry profiles of FITC-labelled OMV isolated from the clinical isolate *M*. *catarrhalis* KR971 (white) as compared to OMV from the MID-deficient *M. catarrhalis* BBH18 Δ*mid* (shaded) when bound to purified tonsilar B lymphocytes. OMV were used at 10 µg/ml and incubated with B cells for 1 h at 37°C. (E) MID expression in OMV isolated from the clinical isolate of *M*. *catarrhalis* was analyzed by SDS-PAGE (Ag-stain) and Western blot (WB) using MID specific rabbit pAbs. Molecular weight markers in kilodalton are indicated to the left.

To confirm that OMV released *in vivo* by *M*. *catarrhalis* also contain MID, a nasal sample from a 9-year old child with newly diagnosed *M*. *catarrhalis* sinusitis was examined by TEM using gold-labelled antibodies directed against MID [9],[42]. Figure 8A shows one *M*. *catarrhalis* bacterium that released OMV containing MID (black arrows) *in vivo*. The presence of DNA associated with these OMV samples was also demonstrated in another bacterium using gold-labelled anti-DNA antibodies (Fig. 8B; white arrows). To ensure that MID and DNA were located on the same OMV, double staining was performed (Fig. 8C). Large granules indicate MID (black arrows), whereas small granules show DNA (white arrows). Thus, each OMV released *in vivo* in a 9-year old child contained the protruding MID molecule in addition to DNA adjacent to the OMV surface.

We also isolated this particular strain, designated *M*. *catarrhalis* KR971, from the child with sinusitis. Importantly, the clinical isolate was only passed once and immediately frozen. To evaluate the capacity of OMV secreted by this isolate to interact with B cells *in vitro*, OMV isolated from an overnight liquid culture were labelled with FITC and incubated with purified tonsillar B lymphocytes. OMV secreted by *M*. *catarrhalis* KR971 clearly bound to B cells when analyzed by flow cytometry, whereas OMV isolated from the isogenic MID-deficient mutant *M*. *catarrhalis* BBH18 Δ*mid*, which were included as a negative control, did not (Fig. 8D). Finally, the presence of MID in OMV released *in vitro* was also confirmed by SDS-PAGE and Western blot using pAbs directed against the IgD-binding region of MID (Fig. 8E).

## Discussion

*Moraxella*-induced T-independent B cell activation is initiated by IgD cross-linking via the superantigen MID [13]. Moreover, PRRs like TLR also play an important costimulatory role in superantigen-dependent B cell activation since signaling via TLR2 and TLR9 is required for a maximal B cell response induced by MID-expressing bacteria [13]. B cell activation induced by *Moraxella* results in the production of polyclonal IgM, and these antibodies are not directed against *Moraxella* suggesting an important role for MID in *M*. *catarrhalis* pathogenesis [14]. We have previously demonstrated that the interaction between MID and IgD also mediates tonsillar B cell endocytosis of whole bacteria. This prompted us to investigate whether tonsillar B cells constitute a potential niche for *M*. *catarrhalis*. However, despite MID expression was required for B cell uptake, *Moraxella* did not survive after it had been endocytosed by tonsillar B cells (Fig. 1). Furthermore, physiological concentrations of soluble IgD did not block the binding of *Moraxella* to human B cells probably due to the high density of MID at the bacterial surface [9],[42]. It is generally accepted that primary B cells are nonphagocytosing cells, albeit recent findings challenge this concept. Souwer *et al*. found that human B cells are able to internalize *Salmonella typhimurium* when bacteria are recognized by the BCR [43], but in contrast what was observed with *Moraxella*, Salmonella survives intracellularly [44],[45]. The exact mechanism how B cells kill *Moraxella* is not clear at present, but fusion of early endosome-containing bacteria with the phagolysosome compartment may be a possibility. The IgD-mediated endocytosis of whole bacteria by tonsillar B cells is a unique uptake mechanism related to the IgD-binding *Moraxella* only. Despite that the uptake might be a disadvantage for the bacteria, *M*. *catarrhalis*-induced B cell proliferation may further increase the number of bacteria that are taken up and processed, but at the same time leading to a polyclonal antibody production with antibodies that are not directed against the bacterial species as such. More studies are required to fully understand the final outcome of this process.

MID is a highly conserved outer membrane protein and the *mid* gene can be detected in essentially all clinical *M*. *catarrhalis* isolates [42]. The IgD-binding domain of MID is only located within 238 amino acids at the distal end of the surface exposed MID molecule, which consists of a total of approximately 2,200 amino acids. Thus, *M*. *catarrhalis* could potentially remove the IgD binding domain and hence the capacity to bind B cells within a few cell generations [8],[9],[42]. Although the exact function of MID-dependent IgD-binding during *Moraxella* pathogenesis still remains elusive, it is most likely that this unique virulence factor plays a significant role in determining the success of *Moraxella* colonization and/or infection.

To further shed light upon the intriguing IgD-binding capacity of *M*. *catarrhalis*, we focused on OMV that are commonly secreted by Gram-negative bacteria including this species. We have previously demonstrated that *Moraxella* OMV can neutralize C3 and hence pave the way for the respiratory pathogen *Haemophilus influenzae* when exposed to the bactericidal effect of human serum [25]. In the present study, we show the presence of MID in secreted *M*. *catarrhalis* OMV both *in vitro* liquid culture of *Moraxella* (Fig. 2) and in a nasal sample from a child newly diagnosed with *M*. *catarrhalis* sinusitis proving the existence of MID-containing OMV *in vivo* (Fig. 8). OMV binding to human tonsillar CD19^+^ IgD^+^ lymphocytes by the superantigen MID results in vigorous activation. The interaction between tonsillar B cells and *Moraxella* OMV induced a phenotypic change in B cells including down-regulation of IgD followed by upregulation of surface antigens. In parallel with the B cell response observed after stimulation with whole *Moraxella*, OMV containing MID induced a significant increase of the IgM production in the absence of physical T cell help or cytokines. No differences in IgG or IgA production were observed after B cell stimulation with OMV isolated from the wild type or the MID-deficient mutant suggesting that OMV activation alone was not enough to drive CSR. In agreement with these results, experiments using a combination of native MID, CD40L, IL-4, and IL-10 only resulted in low IgG and IgA production [2]. The absence of antibody specificity directed against the most dominant respiratory pathogens indicated that the OMV-dependent B cell response may not help the host to clear the respiratory pathogens but suggests that *Moraxella* merely redirects the humoral response that would be beneficial for bacterial colonization. Finally, the strong mitogenic effect on B cells induced by MID-containing OMV in addition to what was previously shown with whole bacteria suggests that pathogen-associated molecular patterns (PAMPs) needed for full B cell activation are present in the OMV [12]–[14]. The main PAMP involved in the B cell activation induced by OMV was bacterial DNA that was associated with OMV and activates the intracellular PRR TLR9. An interesting observation is that several bacterial species including *M*. *catarrhalis* have the capacity to form biofilms and in some cases biofilms are largely built up by OMV [46]. The biofilm offers the bacterium a protective coat against phagocytosis [47]. Another important cornerstone of biofilms is that DNA is secreted during growth and it has been shown that *Pseudomonas aeruginosa* cannot form biofilms when grown in the presence of DNase I [48]. OMV secreted by *Moraxella* during infection contain biologically active molecules which are effective in activating immune host cells and thus represent a potential novel virulence mechanism. It remains, however, to delineate the role of OMV and DNA in *Moraxella* biofilm formation.

Vesicles secreted by *Moraxella* have the capacity to bind tonsillar B cells, and the superantigen MID is responsible for this phenomenon. Binding of OMV followed by internalization into B cells depend specifically on the interaction between MID and surface expressed IgD BCR. TEM analysis with antibodies directed against MID demonstrated that *Moraxella* OMV contained several MID molecules capable of cross-linking multiple IgD BCR needed for B cell stimulation. Several examples of OMV surface factors which mediate adhesion to and invasion of eukaryotic cells have been identified in other pathogens. For example, LPS-bound heat labile enterotoxin (LT) is the adhesin responsible for enterotoxigenic *Escherichia coli* OMV interactions with host cells [22],[49]. *Borrelia burgdorferi*, the spirochete responsible for Lyme disease, produces OMV that bind to endothelial cells through the outer membrane proteins OspA and OspB [23]. Another example is OMV from *Shigella flexneri* that adhere to and enter human intestinal cells *in vitro* via the outer membrane invasins IpaB, C and D [20].

The MID-dependent OMV binding leads to Ca^2+^-mobilization and clustering of receptors in lipid raft motifs in purified B cells. Recent studies demonstrated the role of lipid rafts in BCR-mediated signal transduction (for a review see [32]). The functional significance of lipid raft aggregation induced by OMV was most likely to enhance BCR signaling. Gupta *et al*. [31] have shown that BCR stimulation recruited the tyrosine kinase Syk to lipid rafts and induced concentrated protein tyrosine phosphorylation in the proximity of lipid rafts indicating that BCR signaling is occurring primarily within this compartment. We confirmed the involvement of lipid raft structures in OMV-induced clustering of receptors by testing the effects of filipin, which disrupts lipid motifs. The mobilization of IgD, TLR2, and TLR9 into lipid rafts appeared to be cholesterol dependent, as its localization to specific lipid raft fractions was lost in B cells treated with filipin. A previous study has shown that the association between *E. coli* OMV and host epithelial cells is also sensitive to treatment with filipin [22]. More recently, Bomberger *et al*. demonstrated that *P*. *aeruginosa* OMV deliver multiple virulence factors into host airway epithelial cells via a mechanism of OMV fusion with the lipid raft machinery [24]. In parallel with that study, we suggest in the present paper that OMV-mediated fusion of virulence factors via lipid raft domains is a common strategy of Gram-negative bacteria to interact with the human host.

The signaling through the BCR is noticeably regulated by an array of signaling receptors receiving information from the surrounding milieau of the B cells [50]. In fact, receptors of the innate immune system, in particular TLRs, have shown to influence the result of antigen engagement by the BCR [51],[52]. CpG DNA-induced TLR9 signaling has been demonstrated to synergize with antigen-induced BCR signaling and this synergistic engagement has been implicated in the activation of autoimmune B cells [53],[54]. Recently, Chaturvedi *et al*. [55] demonstrated that the internalized BCR signals recruit TLR9-containing endosomes to the autophagosome and this recruitment showed to be necessary for the B cell hyperresponses. In agreement with these results, we found that OMV induced a strong B cell activation via TLR9 signaling indicating that DNA-containing OMV affect B cell responses and that the modulating effects of TLR9 on downstream BCR signaling events are required for full activation of B cells. After 30 min of incubation with DNA-containing OMV the internalized BCR colocalized TLR9 and Rab5 in early endosomes (Fig. 3D). Relocalization of TLR9 into intracellular structures has been previously shown in mouse B cells after crosslinking of the BCR with anti-IgM pAb conjugated to CpG-containing DNA [55]. These authors proposed a model for B cell hyperresponses to DNA containing antigen in which antigen internalization and BCR signaling recruit TLR9 to autophagosome-like compartments to allow TLR9 to survey the antigen for its DNA ligand. In the case of DNA-containing OMV secreted by pathogenic bacteria, the enhanced signaling through the BCR and TLR9 may contribute to unspecific antibody production by reducing the threshold for B cell activation. Interestingly, we were able to detect both intracellular and extracellular upregulation of TLR9 expression in OMV-stimulated B cells. Surprisingly, no increase in TLR9 transcripts was observed. Regulation of eukaryotic mRNA translation is a fundamental mechanism for moderating cellular events, and micro-RNAs (miRNAs) play important roles in a wide range of biological events through post-transcriptional suppression of target mRNAs. Recent data demonstrate that miRNAs regulate TLR2 and TLR4 expression [56],[57], and the impetus for future studies would be to clarify the post-trancriptional TLR9 regulation in human B cells upon activation through the IgD BCR.

**Figure 9:**
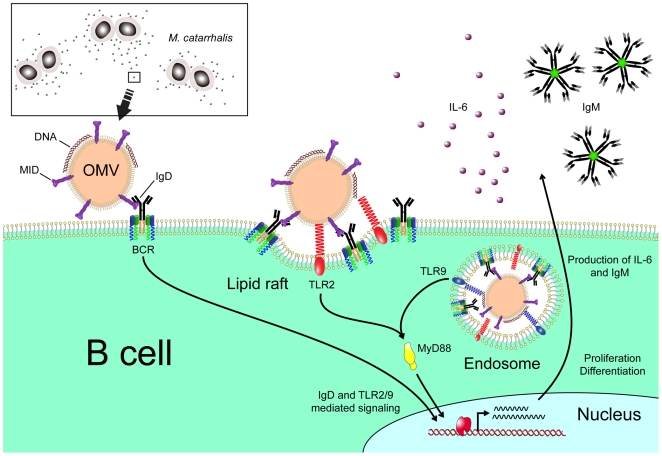
Cartoon schematically showing *M. catarrhalis* OMV-dependent B cell activation. OMV discharged from *Moraxella* are taken up by B cells via an IgD-mediated cross-linking and endocytosis. A signal is induced via the MID-mediated contact with the IgD B cell receptor followed by formation of lipid rafts. In addition, TLR2 colocalize in lipid raft motifs and participate in the signaling induced by OMV. In the endosome, DNA-containing OMV are activating TLR9. The IgD BCR and TLR2/9 mediated signaling results in IL-6 production and eventually IgM secretion.

In conclusion, our results demonstrate that OMV secreted by *M*. *catarrhalis* have the capacity to induce a T cell independent B cell activation by the IgD-binding MID in addition to the PRRs TLR2 and TLR9 (Fig. 9). *Moraxella* OMV can therefore mediate host interactions at a distance from the site of colonization, conferring serum resistance [25] and a delayed specific antibody response not only to *M*. *catarrhalis* but most likely also to other bacterial species such as pneumococci and *H*. *influenzae* that are dwelling in the respiratory tract.

## Materials and Methods

### Antibodies and reagents

Rabbit anti-human IgD, fluorescein isothiocyanate (FITC)-conjugated rabbit anti-human IgM or IgD, R-phycoerythrin (RPE)-conjugated rabbit anti-human IgD polyclonal antibodies (pAbs), RPE-conjugated mouse anti-human CD3, CD14, CD16, CD19, CD45, CD56, CD83 monoclonal antibodies (mAbs), FITC-conjugated mouse anti-human CD19, CD86, CD69 and HLA-DR mAbs were purchased from DAKO (Glostrup, Denmark). Mouse anti-human TLR9 mAbs were supplied by InvivoGen (San Diego, CA). FITC-conjugated mouse anti-human TLR9 and anti-human TLR2 mAbs were obtained from Imgenex (San Diego, CA). Mouse anti-flotillin-2, mouse anti-caveolin-1 and mouse anti-Rab5 mAbs were purchased from BD Bioscience (San Diego, CA). The truncated recombinant MID^962-1200^ and MID^1000-1200^ fragments, and rabbit anti-MID^962-1200^ antiserum were prepared as described earlier [10],[58]. Filipin III was purchased from Sigma-Aldrich (St. Louis, MO). The TLR ligands CpG ODN 2006 and the suppressive oligonucleotide (ODN) with human-specific CpGs (TTAGGG)×4 were supplied from Invivogen. Alexa Fluor 488 goat anti-mouse IgG, Alexa Fluor 594 goat anti-mouse IgG, Alexa Fluor goat anti-mouse 633, Alexa Fluor 546 goat anti-rabbit IgG and ProLong Gold antifade reagent with DAPI (4′, 6-diamidino-2-phenylindole) were purchased from Molecular Probes (Invitrogen, Carlsbad, CA). Purified IgD from human serum was obtained by affinity chromatography as described earlier [59].

### Bacterial strains and growth conditions

The *M*. *catarrhalis* strains used in this study are described in Table 1. Bacteria were routinely cultured in brain heart infusion (BHI) liquid broth or on BHI agar plates at 37°C. *M*. *catarrhalis* BBH18 mutants were previously described [42],[58],[60]. The MID-deficient mutants were cultured in BHI supplemented with 50 µg/ml kanamycin. A set of UspA1 and A2 deficient mutant was also included as controls. The UspA1-deficient mutant was cultured in BHI supplemented with 1.5 µg/ml chloramphenicol (Sigma-Aldrich), and the UspA2-deficient mutant was incubated with 7 µg/ml zeocin (Invitrogen). Both chloramphenicol and zeocin were used for growth of the UspA1/A2 double mutants. Genomic *M*. *catarrhalis* DNA from the BBH18 wild type strain was extracted using the DNeasy kit (Qiagen, Hilden, Germany) according to the manufacturer's recommendations. Purified DNA was free of contaminants and enzyme inhibitors.

### Isolation of outer membrane vesicles and DNA analysis

OMV were prepared from overnight cultures according to the Rosen method [61]. Briefly, cell free supernatants were filtered through a 0.2 µm-pore size filter (Sartorius, Epson, UK) and concentrated using 100 kDa Vivaspin centrifugal concentrators (Vivascience, Hannover, Germany). The concentrated supernatants were thereafter centrifuged at 100,000×g for 60 min. The precipitates containing OMV were washed three times in PBS followed by a final sterile filtration to exclude cellular contamination from the parent OMV-producing *M*. *catarrhalis*. Protein content was determined by spectrophotometry using NanoDrop (NanoDrop Technologies, Wilmington, DE). The purity of OMV samples was examined by transmission electron microscopy (TEM) and by excluding bacterial growth of any remaining parent cells on BHI agar. To evaluate a putative uptake of exogenous DNA encapsulation by OMV, a DNase assay was performed as previously described by Renelli *et al*. [21]. Briefly, two Ehrlenmeyer flasks were inoculated with *M*. *catarrhalis* BBH18, one was the control and one was supplemented with a final concentration of 100 µg DNase/ml (Sigma) and 10 mM MgCl_2_ (“OMV DNase culture”). Flasks were incubated at 37°C (200 r.p.m.) until late exponential phase followed by OMV isolation. The uptake of DNA within OMV in the presence of DNase was examined by PCR and TEM as described below. DNA extraction using DNeasy® Blood & Tissue kit (Qiagen) was performed on untreated OMV and DNase treated OMV. The final ultracentrifuge supernatant (free of bacteria and OMV) from both cultures was analyzed to determine whether extracellular DNA was present. Whole cell genomic DNA was included as a positive control. Samples containing DNase were heated at 100°C for 15 min before being analyzed by PCR according to a standard protocol. DNA primers used for the amplification of the *M*. *catarrhalis* 16S rRNA gene were 5′-GCCCTGACGTTACCCACA-3′ and 5′-TCACCGCTACACCTGGAA-3′. PCR amplification consisted of a 3 min hot start of 95°C followed by 30 cycles of 45 sec. at 95°C, 30 sec. at 54°C and 15 sec. at 72°C. The reaction was completed with an extension step of 5 min at 72°C. In experiments with DNase-treated OMV, preparations were treated with 50 µg/ml DNase I (Sigma) in 10 mM MgCl_2_ to digest putative DNA bound to the outer surface of OMV. After incubation for 1 h at 37°C, the OMV were washed twice with PBS by ultracentrifugation followed by heat inactivation at 100°C.

### SDS-PAGE and Western blots

The protein content of OMV was analyzed on a 10% SDS-PAGE stained with BioRad Silver Stain Plus kit (Munich, Germany). Proteins were transferred at 20 V overnight to an Immobilon-P membrane (Millipore, Bedford, MA). After transfer, the membranes were blocked for 2 h using PBS with 0.1% Tween (PBS-Tween) and 5% skim milk powder. After several washes with PBS-Tween, the membrane was incubated with a rabbit anti-MID^962-1200^ antiserum as described [10],[58]. Repeated washes with PBS-Tween were followed by incubation with horseradish peroxidase (HRP)-conjugated goat anti-rabbit pAbs (Dakopatts, Copenhagen, Denmark) in PBS-Tween including 2% skim milk for 45 min. The transferred proteins were detected using ECL Western blot detection reagents (Amersham Pharmacia Biotech, Uppsala, Sweden).

### B cell isolation

Tonsils (*n* = 16) were obtained from patients under the age of 12 (age range: 3–12 years old) undergoing tonsillectomy at the University Hospital in Malmö. The Ethics Committee of Lund University approved the study (No. 877/2005) and a signed written informed consent was obtained from the parents (or legal representatives) of all patients. Surgery was performed due to tonsillar hyperplasia and apart from the tonsillar symptoms, all patients were healthy and did not receive any medication. Tonsils were dissected in RPMI 1640 medium supplemented with 10% FCS, 2 mM glutamine, 50 µg/ml gentamicin and 100 U/ml penicillin (complete medium). The homogenized cell suspensions were filtered through a 70 µm nylon cell strainer (Becton Dickinson, NJ) followed by isolation of lymphocytes on Lymphoprep® (Nycomed, Oslo, Norway) density-gradients as described [10]. Untouched CD19^+^ B cells were isolated by an indirect magnetic labelling system (B Cell Isolation Kit II; Miltenyi Biotec, Bergisch Gladbach, Germany) with an additional purification step using magnetic labelled antibodies directed against CD3 (Miltenyi Biotec) resulting in an ultrapure B cell preparation (99%≥CD19^+^). B cells were routinely screened for contaminating monocytes, T cells, NK cells, or dendritic cells using RPE-conjugated mAbs against CD3, CD14, CD16, CD56, or CD83 in combination with FITC-conjugated anti-human CD19 mAbs after isolation and during culture. In all experiments with lymphocytes, 1×10^6^ cells/ml were cultured in complete medium supplemented with various reagents in culture plates (Nunc, Roskilde, Denmark). OMV from BBH18 strains were added at concentrations ranging from 0.1–25 µg/ml. The TLR ligand CpG ODN 2006 and the dominant negative TLR9 ligand (TTAGGG)×4 were added at 1 µM and 8 µM, respectively. The IgD binding part of MID (MID^962-1200^) was as previously described [58] and coated in Tris-HCl buffer (pH 9.0) overnight. Proliferation was measured by [methyl-^3^H]-thymidine incorporation (1 µCi/well, Amersham Pharmacia Biotech) using an 18 h pulse period. B cell viability after each treatment was routinely assessed by trypan blue exclusion staining.

### Intracellular survival assay

To examine bacterial intracellular survival on purified tonsillar B lymphocytes, we used a modification of the method described by Slevogt *et al*. [62]. *M*. *catarrhalis* BHH18 wild type or the corresponding mutant BBH18 Δ*mid* were resuspended in PBS and added to 1×10^5^ B cells at multiplicity of infection (MOI) of 100 followed by incubation at 37°C and 5% CO_2_ for 1 h. After several washes, infected B cells were incubated in RPMI 1640 medium containing 200 µg/ml gentamicin for 1 h to kill extracellular bacteria. Subsequently, infected B cells were washed three times with PBS and resuspended in RPMI 1640 medium followed by incubation at 37°C. The number of viable intracellular bacteria was determined after 0–4 h of further incubation. To lyse the lymphocytes and release *M*. *catarrhalis*, infected cells were resuspended in 1 ml PBS and transferred to a glass tube containing glass pearls to mechanically lyse the cells by vigorous vortex for 1 min. An aliquot of lysed cells was serially diluted and quantitatively plated on BHI agar plates. Colony forming units (cfu) were counted after 24 h of incubation at 37°C. No changes in the number of viable lymphocytes were detected upon infection. Control experiments to assess efficacy of antibiotic bactericidal activity were performed in parallel. Briefly, samples of 1×10^8^ bacteria were incubated with RPMI 1640 medium containing gentamicin and plated on BHI agar after 1 h at 37°C. This treatment resulted in complete killing as judged by colony forming units (cfu).

### Measurement of Ca^2+^ mobilization

Ca^2+^ mobilization was measured with Fura-2 according to the following protocol. Purified lymphocytes were resuspended at a density of 10^7^/ml in HBSS (Hank's balanced salt solution)-1% BSA buffer (Gibco, Invitrogen Cell Culture) and incubated in a waterbath at 37°C in the presence of 2 µM Fura-2 AM (Molecular Probes, Eugene, OR), followed by two washes in the above medium. The Fura-2 loaded cells were then resuspended at a density of 2×10^6^/ml in a optical methacrylate (PMMA) disposable cuvettes (Kartell; Merck, Poole, UK) in 2 ml of HBSS-1% BSA buffer. Ca^2+^ mobilization into the cytosol was monitored at 340 and 380 nm (excitation) and 510 nm (emission) with a spectrofluorimeter (FluoroMax, Spex Industries, Edison, NJ) using the dM3000 Software. Ca^2+^ concentrations were calculated using the Grynkiewicz equation [63]. For the stimulation of B cells, OMV (10 µg/ml) and ionomycin (100 nM) (Sigma-Aldrich) were added at 50 sec. after the start of data acquisition.

### Isolation of lipid rafts

A modification of the method of Brown *et al*. was used to isolate lipid rafts [64]. Tonsillar B cells were exposed to *M*. *catarrhalis* (1×10^7^ CFU/ml) or OMV (10 µg/ml) for 30 min and/or filipin 20 µg/ml for 30 min prior to stimulation. The cells were washed with cold PBS and lysed with 0.5% Triton X-100 in TNE buffer (25 mM TrisCl, pH 7.5, 150 mM NaCl, and 5 mM EDTA) plus protease inhibitors for 20 min on ice. Lysed cells were harvested and homogenized with a loose-fitting followed by a tight-fitting Dounce homogenizer. Whole cells and nuclei were removed by centrifugation at 1,000×g for 10 min. An equal volume of 90% sucrose in TNE buffer was added to the supernatant. This 45% layer was overlaid with 30% and 5% sucrose in TNE buffer to form a discontinuous gradient. Samples were centrifuged at 200,000×g for 18 h at 4°C followed by collection of 1 ml fractions. Protein concentrations were determined using a NanoDrop and 5 µg was analyzed on a 12% SDS-PAGE. After transfer to PVDF Immobilon-P (Millipore), blots were incubated in 5% skim milk blocking solution for 1 h at room temperature (RT). HRP-conjugated anti-goat or anti-mouse IgG (DAKO) and Western Lightning Chemiluminescence Reagent Plus (Perkin Elmer Life Sciences, Boston, MA) were used for visualization.

### Flow cytometry analyses

Surface expression of IgD and IgM after addition of OMV was monitored using flow cytometry (Becton Dickinson, Franklin Lakes, NJ). Purified B cells were incubated in complete medium with different concentrations of OMV ranging from 0.625–10 µg/ml and analyzed for IgM and IgD expression at various time points (0–24 h). Harvested cells were washed and incubated in PBS containing 2% BSA with FITC-conjugated rabbit anti-human IgM and RPE-conjugated rabbit anti-human IgD pAb for 1 h on ice. After two washes in PBS, B cells were screened for IgM and IgD density using flow cytometry. The internalization of IgD after stimulation with OMV was also examined by flow cytometry. Purified tonsillar B cells were stimulated with wild type or MID-deficient OMV, fixed and incubated with FITC-conjugated anti-CD19 mAb and rabbit anti-IgD pAb to block surface expressed BCR. After several washes, B cells were incubated in permeabilization buffer (0.03% Triton X-100 and 5% normal serum blocking solution in PBS) followed by incubation with RPE-conjugated anti-IgD mAb. The B cell surface phenotype changes after exposure to OMV was also investigated using flow cytometry analysis. Stimulated B cells were harvested in PBS-BSA and labelled with antibodies directed to different surface markers for 1 h on ice. For TLR9 screening, B cells stimulated with OMV were fixed and stained with RPE-CD19 mAb. After several washing steps, cells were incubated in permeabilization buffer (0.03% Triton X-100 and 5% normal mouse serum blocking solution in PBS) before adding FITC-conjugated anti-TLR9 mAbs. For extracellular TLR9 screening, B cells stimulated with *Moraxella* OMV were fixed and stained with FITC-conjugated anti-TLR9 mAbs.

The ability of purified human IgD to block *M*. *catarrhalis* binding to B cells was also tested by flow cytometry experiments. FITC-labelled *M*. *catarrhalis* wild type were treated with increased concentration of purified human IgD (0–150 µg/ml) for 1 h. After several washes, bacteria were incubated with purified human B cells for 30 min at 37°C followed by washes. Bacterial binding to B cells were assessed by flow cytometry. In order to corroborate that the purified human IgD bound to FITC-labelled *M*. *catarrhalis*, bacteria were incubated with human IgG standard (50 µg/ml) or purified IgD (50 µg/ml). After several washes, bacteria were incubated with an RPE-conjugated anti-human IgD mAb and analyzed by flow cytometry.

Binding of FITC-stained OMV to B cells was also tested. Briefly, 2×10^5^ purified B cells were incubated with 10 µg/ml FITC-stained OMV from *M*. *catarrhalis* wt or Δ*mid* for 1 h at 37°C. B lymphocytes were washed twice with PBS before analysis. To distinguish between intracellular and extracellular FITC-stained OMV, we used a fluorescence quenching method as described [35]. The quenching dye was prepared as follows: trypan blue (2 mg/ml; Merck) was dissolved in 0.15 M NaCl in 0.02 M NaAc buffer (pH 4.4) containing crystal violet (2 mg/ml; Merck). The quenching dye was added to the cells previously incubated with FITC-stained OMV directly before flow cytometry analysis.

### Cytokine protein Array, IL-6 and Ig ELISA

To analyse cytokine production, 1×10^6^ purified tonsillar B cells were cultured with or without 10 µg/ml OMV in 12-well flat-bottom plates (Nunc-Immuno Module) in a final volume of 1 ml complete medium. The cell-free supernatant was harvested after 96 h, and the cytokine protein arrays were performed according to the manufacturer's instructions (RayBio Human Cytokine Antibody Array, RayBiotech, Norcross, GA). IL-6 production was determined using enzyme-linked immunosorbent assay (ELISA) plates from R&D systems (Minneapolis, MN). IgG, IgA and IgM were measured in cell free B cell supernatants harvested after 96 h. ELISA plates (Maxisorb, Nunc) were coated at 4°C overnight with rabbit anti-human IgG, IgA or IgM pAb (Dakopatts) in 0.1 M Tris-HCl (pH 9.0). The plates were washed four times with PBS-Tween followed by 2 h blocking at RT using PBS-Tween supplemented with 1.5% ovalbumin HRP-conjugated rabbit anti-human IgG, IgA or IgM (Dakopatts) pAb, respectively, that were used as detection antibodies. Finally, the plates were developed and measured at OD_450_. A standard serum (Dakopatts) was included for calculation of the Ig concentrations.

### Fluorescence, confocal and transmission electron microscopy

For fluorescence and confocal microscopy, B cells (1×10^6^) were incubated with OMV (10 µg/ml) in 1 ml complete medium for 30 min at 37°C. After several washes with ice cold phosphate-buffered saline (PBS), cells were fixed with 4% paraformaldehyde solution for 10 min at RT, washed and incubated with 5% normal serum blocking solution for 20 min at RT. Primary antibodies were added for 1 h at RT followed by three washes. For TLR9 staining, cells were fixed and incubated in permeabilization buffer (0.03% Triton X-100 and 5% normal serum blocking solution in PBS) before adding anti-TLR9 mAbs. Alexa Fluor-conjugated secondary antibodies were incubated in the dark for 1 h at RT. For the triple staining procedure, stimulated B cells were fixed, permeabilized as described before and incubated with rabbit anti-IgD pAb (BCR) and mouse anti-Rab5 mAb (Rab5) for 1 h at RT. After several washes, B cells were incubated with Alexa Fluor 633 goat anti-mouse IgG and Alexa Fluor 546 goat anti-rabbit IgG secondary mAb followed by incubation with FITC-conjugated anti-TLR9 mAb (TLR9). After washing twice with PBS, cells were resuspended in 50 µl of PBS, of which 15 µl was transferred to polylysin glass slides and allowed to sediment for 15 min at RT. The cells were fixed to the glass surface with DAPI containing Prolong Gold antifade reagent overnight and examined by immunofluorescence microscopy or by confocal microscopy using a Bio-Rad Radiance 2000 confocal system fitted on a Nikon microscope with a×60/NA 1.40 oil lens. B lymphocytes showing polarization of lipid rafts were counted by microscopic examination of 25 randomly selected fields, showing a minimum of three B cells per field. Cells showing signs of clustering were expressed as percentage of the total number of cells per field.

For immunohistochemistry and TEM, B cells (1×10^6^) were incubated with 5×10^7^ *M*. *catarrhalis* wild type in 1 ml complete medium for 1 h at 37°C, followed by centrifugation and fixation in TEM fixative. Alternatively, bacteria from cultures untreated or DNase treated were centrifuged and fixed. A fresh nasal discharge from a 9-year-old child with *M*. *catarrhalis* sinusitis (pure growth of *Moraxella* on nasal aspirate culture) was also examined. This was prepared by suspending a drop of the purulent nasal discharge in 1 ml of PBS with 4% paraformaldehyde. The cellular fraction was obtained by centrifuging the specimen at 214×g. Aliquots were thereafter examined by TEM. Ultrathin sections were mounted on gold grids and subjected to antigen retrieval using sodium metaperiodate [65],[66]. For immunostaining, the grids were floated on top of drops of immune reagents displayed on a sheet of parafilm. Free aldehyde groups were blocked with 50 mM glycine, and the grids were then incubated with 5% (vol/vol) goat serum in incubation buffer [0.2% bovine serum albumin-C in PBS, pH 7.6] for 15 min. This blocking procedure was followed by overnight incubation with primary antibodies (dilution 1∶50–1∶100) at 4°C. After washing the grids in 200 µl incubation buffer, floating on drops containing the gold conjugate reagents (diluted 1∶10–1∶20 in incubation buffer) was performed for 60 min at RT. The sizes of the gold particles used were 10 and 5 nm. After further washes in incubation buffer, the sections were postfixed in 2% glutaraldehyde. Finally, sections were washed in distilled water and poststained with uranyl acetate and lead citrate and examined under the electron microscope. Specimens were observed in a Jeol JEM 1230 electron microscope (JEOL, Tokyo, Japan) operated at 60 kV accelerating voltage. Images were recorded with a Gatan Multiscan 791 CCD camera (Gatan, Pleasanton, CA).

### RNA isolation and quantitative real-time reverse transcription-polymerase chain reaction (RT-PCR)

Stimulated B cells were lysed in RLT buffer (Qiagen, Hilden, Germany), supplemented with 1% 2-mercaptoethanol, and stored at −80°C until use. RNA was extracted using an RNeasy Mini Kit (Qiagen). The quantity and quality of the RNA was determined by spectrophotometry using the A_260/280_ ratio. The Omniscript Reverse Transcriptase kit (Qiagen) and Oligo(dT)15 primer (Novagen, Nottingham, UK) were used for first-strand cDNA synthesis with an aliquot of 20 ng RNA as starting material. The resulting cDNA was diluted with water and 18 ng was used for amplification. The RT-PCR was performed on a Smart Cycler (Cepheid, Sunnyvale, CA) using TaqMan Universal PCR Master Mix, No AmpErase UNG and Assay-on-Demand Gene Expression products (Applied Biosystems, Foster City, CA), containing unlabelled primers and MGB probe (FAM™ dye-labelled). The thermal cycler was programmed to perform an initial set-up (95°C, 10 min) and 45 cycles of denaturation (95°C, 15 seconds) followed by annealing/extension (60°C, 1 min). The relative amounts of mRNA for TLR9 were determined by subtracting threshold (Ct) values for these genes from the Ct value for the internal control gene β-actin (ΔCt). Data were depicted as 2^ΔCt^×10^5^ and presented as mean values±standard error of the mean (SEM).

### Statistical analysis

Statistical analysis was performed using GraphPad PRISM 5 (San Diego, CA) and significance was calculated with one-way repeated measures ANOVA with Dunett's multiple comparison test (for comparisons of more than two data sets). The Student's t-test was used to determine statistical differences for unpaired comparisons with Welch correction if variances were non-homogenous. Significant values were defined as *, *p*≤0.05; **, *p*≤0.01. All data are expressed as mean±SEM, and *n* corresponds to the number of experiments performed.

### Accesion numbers

MID/Hag (AAX56613).
